# Identification of Key Candidate Genes Potentially Associated with Lactation Traits in Dairy Cows Using Weighted Gene Co-Expression Network Analysis

**DOI:** 10.3390/vetsci13070693

**Published:** 2026-07-16

**Authors:** Tong Mu, Zhixuan Qiao, Honghong Hu, Yun Ma, Ziyan Jiang, Yuxin Huang, Zhihong Sun

**Affiliations:** 1 Key Laboratory of Ruminant Molecular Cell Breeding in Yan’an, College of Life Sciences, Yan’an University, Yan’an 716000, China; 18303418951@163.com (Z.Q.); h918622@163.com (H.H.); 13689282839@163.com (Z.J.); 18434482643@163.com (Y.H.); sunzhihong@yau.edu.cn (Z.S.); 2 College of Life Sciences, Ningxia University, Yinchuan 750021, China; 3 Key Laboratory of Genetic Breeding and Efficient Utilization of Featured Livestock and Poultry Resources in Ningxia, College of Animal Science and Technology, Ningxia University, Yinchuan 750021, China; tmlf74@126.com

**Keywords:** Holstein dairy cow, lactation traits, WGCNA, genes

## Abstract

Lactation traits are important indicators for evaluating the production performance of dairy cows and the economic efficiency of dairy production. Identifying potential regulatory genes is essential for elucidating the molecular mechanisms underlying lactation and facilitating molecular breeding. This study aimed to identify candidate genes and regulatory pathways potentially associated with lactation traits in dairy cows by integrating transcriptome expression profiles of primary bovine mammary epithelial cells (BMECs) from eight Holstein cows with lactation phenotypic data. A gene co-expression network was constructed using weighted gene co-expression network analysis (WGCNA). Co-expression modules associated with lactation traits were identified, and candidate genes were further screened by integrating gene significance, module membership, functional enrichment analysis, random forest analysis, gene-phenotype association analysis, single-gene gene set enrichment analysis (GSEA), ROC curve analysis, and tissue expression profiling. Four co-expression modules, namely MEdarkturquoise, MEsteelblue, MEbrown, and MEskyblue3, were significantly associated with lactation traits (*p* < 0.05). Genes in these modules were mainly enriched in biological processes and pathways related to ribosome biogenesis, protein translation, the cell cycle, oxidative phosphorylation, and the PI3K-Akt signaling pathway, which may be involved in lactation regulation. Through multi-strategy cross-screening, four candidate genes were ultimately identified. *MDM*2 was associated with daily milk yield (DYM), *RPL37*A with total milk solids (TMS), and *SLC25A*25 and *CCNE*2 with milk fat percentage (MFP) and fat-to-protein percentage ratio (FPP). Reverse transcription quantitative real-time PCR (RT-qPCR) validation showed that *MDM*2 and *RPL37A* were relatively highly expressed in mammary tissue, suggesting that they may be involved in lactation-related biological processes in dairy cows. These findings provide potential candidate regulators and theoretical support for further studies on the molecular mechanisms of lactation traits; however, their functional roles require further validation through in vitro and in vivo experiments.

## Simple Summary

Milk production traits, including milk yield and milk composition, are important indicators of dairy cow productivity and farm profitability. However, the biological mechanisms underlying variation in these traits remain incompletely understood. This study aimed to identify potential genes associated with lactation performance by integrating gene expression profiles from bovine mammary epithelial cells of Holstein cows with their milk production records. We identified several groups of genes that were associated with milk yield, milk fat percentage, the balance between milk fat and protein, and total milk solids. These genes were mainly involved in fundamental biological processes, including protein synthesis, cell proliferation, energy metabolism, and signaling pathways. Through integrated screening, four promising candidate genes were identified: *MDM*2, *RPL37*A, *SLC25A*25, and *CCNE*2. These findings provide useful insights into the biological regulation of lactation traits in dairy cows and may support future molecular breeding strategies aimed at improving dairy production efficiency.

## 1. Introduction

With rising living standards and increasing demand for dairy products, improving milk yield and milk quality in dairy cows has become a key objective for the high-quality development of the modern dairy industry. Milk contains abundant fat, protein, lactose, minerals, and vitamins, and represents an important source of high-quality animal protein and essential nutrients for humans [1,2,3,4]. Previous studies have predicted that global demand for milk will increase by approximately 30% by 2030 [5]. Therefore, breeding dairy cows with high yield and superior milk quality is essential for ensuring a stable dairy supply and promoting the sustainable development of the dairy industry [6].

Lactation traits in dairy cows are closely associated with milk production performance and milk quality and are key determinants of their economic value [7]. Previous studies have shown that environmental and physiological factors, including calving season, parity, and lactation stage, significantly affect lactation traits [8,9,10,11]. Genetic factors also play a critical role in the formation of these traits. For example, the K232A mutation in the DGAT1 gene has been widely confirmed to be significantly associated with milk fat percentage in dairy cows [12,13,14]. However, lactation traits are typical complex quantitative traits, generally regulated by the cumulative effects of multiple minor genes and gene-gene interactions. Traditional single-gene approaches are insufficient to systematically reveal their genetic basis at a systems level. These limitations highlight the need to identify candidate genes potentially associated with lactation traits and to explore the regulatory networks underlying lactation in dairy cows.

Weighted gene co-expression network analysis (WGCNA) is an important systems biology approach for constructing co-expression networks based on gene expression profiles [15]. It groups genes with similar expression patterns into functional modules and evaluates the associations between these modules and target traits, thereby facilitating the identification of hub genes and biological pathways related to the traits [16,17,18,19]. WGCNA has been widely applied to the study of economically important traits in livestock species such as pigs and sheep, leading to the identification of multiple functional genes involved in growth and development, reproduction, and productive performance [15,20,21,22,23]. In transcriptomic studies of mammary tissue from Holstein cows, WGCNA has also been used to identify important candidate genes associated with milk yield and milk composition [23,24,25,26]. In addition to WGCNA-based studies, previous transcriptomic analyses have identified several genes and pathways potentially involved in milk production and milk composition traits in dairy cattle, including genes related to lipid metabolism, protein synthesis and mammary gland development. These studies have provided valuable insights into the molecular basis of lactation regulation [27]. Nevertheless, the genetic regulatory basis of lactation traits in dairy cows remains complex, and key genes and regulatory modules associated with these traits have not yet been fully characterized. Furthermore, previous transcriptomic studies of lactation traits in dairy cattle still have several limitations. Many have focused on a limited number of traits or prioritized candidate genes mainly based on differential expression, module membership, or intramodular connectivity.

Given that lactation performance is a complex phenotype involving milk yield and multiple milk composition traits, a more integrated analytical framework is needed to improve candidate gene prioritization. To achieve this aim, RNA-seq expression profiles of primary BMECs collected from Holstein dairy cows during the mid- and late-lactation stages [28] were used together with matched phenotypic records, including age and lactation-related traits such as days in milk (DIM), daily milk yield (DYM), milk fat percentage (MFP), milk fat yield (MFY), milk protein percentage (MPP), milk protein yield (MPY), milk lactose percentage (MLP), total milk solids (TMS), fat-to-protein percentage ratio (FPP), and somatic cell count (SCC). WGCNA was performed to construct a gene co-expression network and identify modules significantly associated with lactation-related traits. Candidate genes were further prioritized through functional enrichment analysis, protein–protein interaction (PPI) network analysis, random forest analysis, gene-phenotype association analysis, single-gene gene set enrichment analysis (GSEA), and ROC curve analysis. Compared with previous studies relying on a single analytical approach, the stepwise analytical pipeline applied in the present study integrates multiple lines of evidence to improve the reliability and biological interpretability of candidate gene prioritization and to identify candidate genes for future functional validation and molecular marker development.

## 2. Materials and Methods

### 2.1. Data Sources

The expression profiles used in this study were derived from transcriptome sequencing data of primary BMECs previously generated by our research group [29]. In the previous study, eight Holstein cows with extreme differences in MFP were selected from 245 second-parity Holstein cows in the mid-to-late lactation stage (150–220 days in milk), based on dairy herd improvement (DHI) records collected from the farm over the preceding year. These cows were similar in age (29–31 months), were all second-parity Holstein cows in the mid-to-late lactation stage, and were maintained on the same farm under identical feeding, housing, and management conditions. All cows were fed the same total mixed ration (TMR), and the dietary composition and nutrient levels are shown in Table 1. They were housed under the same barn conditions and managed according to standard procedures for lactating dairy cows, including consistent feeding, milking, drinking water supply, and daily care. Although the sample size was relatively small, these eight cows were selected from a larger population with clear phenotypic differences in MFP, while major confounding factors such as parity, age, lactation stage, feeding conditions, and management environment were strictly controlled. Therefore, these samples provided a controlled dataset for exploratory transcriptomic analysis of lactation-related traits (Table 2). Nevertheless, given the limited sample size, the WGCNA results should be interpreted as exploratory and hypothesis-generating rather than definitive evidence of causal regulatory relationships.

Fresh milk samples were aseptically collected at three daily milkings, namely morning, noon, and evening, and mixed at a ratio of 4:3:3 for the determination of DHI indicators. In parallel, aseptically collected milk samples were placed into 50 mL centrifuge tubes, sealed, and transported to the laboratory at ambient temperature for the isolation and culture of primary BMECs. Library construction and transcriptome sequencing were subsequently performed by Novogene Bioinformatics Technology Co., Ltd. (Beijing, China). Briefly, total RNA was extracted from primary BMECs using the TRIzol method. RNA degradation and contamination were assessed using 1% agarose gel electrophoresis, RNA purity was evaluated with a NanoPhotometer^®^ spectrophotometer (IMPLEN, Munich, Germany), and RNA integrity was determined using the RNA Nano 6000 Assay Kit on the Bioanalyzer 2100 System (Agilent Technologies, Santa Clara, CA, USA). The 260/280 ratios of all samples ranged from 1.70 to 1.90, and the RNA integrity number (RIN) values were ≥8.00. Ribosomal RNA was removed using the Epicentre Ribo-Zero™ rRNA Removal Kit, and linear RNA was digested with RNase R. Sequencing libraries were constructed using the NEBNext^®^ Ultra™ Directional RNA Library Prep Kit for Illumina^®^ (NEB, Ipswich, MA, USA) according to the manufacturer’s instructions. After library quality control, paired-end 150 bp sequencing was performed on an Illumina platform. After filtering the raw reads, clean reads were aligned to the bovine reference genome ARS-UCD1.2 using Bowtie2 software (v2.2.8).

### 2.2. Overall Analytical Workflow and Rationale

To improve the biological interpretability and reliability of candidate gene identification, a stepwise filtering strategy was applied. First, WGCNA was used to identify gene co-expression modules significantly associated with lactation-related traits, thereby narrowing the analysis to trait-relevant gene sets. Second, PPI network analysis was performed to identify genes with potential central roles in protein interaction networks, as highly connected genes may have important biological functions. Third, random forest analysis was used as a machine-learning approach to prioritize genes with strong discriminatory or predictive importance. Fourth, correlation analysis was conducted to evaluate the associations between candidate gene expression and lactation phenotypes, ensuring their relevance to the traits of interest. GSEA was then used to explore the potential biological pathways and functional processes associated with these genes. ROC analysis was applied to assess the ability of selected genes to distinguish different trait-related groups. Finally, reverse transcription quantitative real-time PCR (RT-qPCR) validation was performed to confirm whether the candidate genes were expressed in lactation-related tissues and to support their potential biological relevance. This sequential strategy was designed to progressively identify candidate genes that were not only statistically associated with lactation traits but also biologically interpretable and potentially relevant to lactation regulation.

### 2.3. Weighted Gene Co-Expression Network Analysis

The standardized gene expression matrix was analyzed using the WGCNA package (https://cran.r-project.org/web/packages/WGCNA/index.html, accessed on 2 January 2026) in R software (R version 4.4.1; https://www.r-project.org/; accessed on 2 January 2026). Before network construction, the median absolute deviation (MAD) was calculated for each gene using the apply function in R to assess expression variability across samples. Genes with low variability were removed according to the criterion MAD > max (Q1, 1.3), where Q1 represents the first quartile, namely the 25th percentile, of the MAD distribution across all genes. The goodSamplesGenes function in the WGCNA package was then used to check for genes and samples with excessive missing values or those failing quality-control criteria. After this variation-based filtering and quality-control procedure, 10,568 genes with relatively high expression variability were retained for subsequent WGCNA.

Subsequently, the gene expression data were then matched with lactation trait phenotype data to construct a gene-trait correlation matrix. The soft-thresholding power was selected using the pickSoftThreshold function in the WGCNA package. The optimal power was determined by comprehensively considering the scale-free topology fit index and mean connectivity. Although the scale-free topology fit index did not reach 0.85, the selected power yielded a relatively high fit index (R^2^ = 0.78) while maintaining sufficient network connectivity, and was therefore considered appropriate for subsequent network construction. Based on this threshold, a topological overlap matrix (TOM) was constructed, and gene modules were identified using the dynamic tree cut algorithm. To reduce redundancy among highly similar modules, modules with eigengene correlation greater than 0.75, corresponding to a module eigengene dissimilarity cut height of 0.25, were merged according to the commonly used WGCNA procedure. Modules significantly associated with phenotypes were identified by calculating correlations between module eigengenes and traits (*p* < 0.05). In addition, module membership (MM) and gene significance (GS) were calculated for genes within each module. Genes with GS ≥ 0.4 and MM ≥ 0.9 [20] were defined as hub genes, and the top 50 genes ranked by connectivity were selected as core candidate genes.

### 2.4. Screening of Core Genes

Based on the STRING database (https://string-db.org/; accessed on 3 January 2026), PPI networks were constructed for genes within modules significantly associated with lactation traits, and the results were imported into Cytoscape software (https://cytoscape.org/; accessed on 3 January 2026). The local average connectivity (LAC) score of each gene was calculated using the CytoHubba plugin in Cytoscape (https://apps.cytoscape.org/apps/cytohubba; accessed on 5 January 2026). LAC was selected as the node importance indicator because it reflects the local topological importance of a node by evaluating the connectivity among its neighboring nodes. Specifically, LAC measures the density of connections within the neighborhood of a given node, thereby facilitating the identification of genes located in highly interconnected functional subnetworks. Genes ranked in the top 50% by LAC score were selected for subsequent analysis, based on the method implemented in CytoHubba [30]. Subsequently, these genes were intersected with hub genes identified based on GS and MM thresholds and with the top 50 genes ranked by connectivity in the WGCNA to identify module-specific core genes. Finally, Venn diagrams were generated using the venn.diagram function in R software (https://www.r-project.org/; accessed on 10 January 2026).

### 2.5. Functional Enrichment Analysis

Gene Ontology (GO) functional enrichment and Kyoto Encyclopedia of Genes and Genomes (KEGG) pathway enrichment analyses of the core genes in each module were conducted using the KOBAS online analysis platform (http://kobas.cbi.pku.edu.cn/; accessed on 4 February 2026). GO annotations were based on the Gene Ontology database (http://geneontology.org/; accessed on 6 February 2026), and pathway annotations were obtained from the KEGG database (https://www.kegg.jp/; accessed on 6 February 2026) integrated in KOBAS. The annotated genes of Bos taurus in KOBAS were used as the reference background for enrichment analysis. GO terms and KEGG pathways with *p* < 0.05 were considered significantly enriched. The enrichment results were visualized using R software (https://www.r-project.org/; accessed on 8 February 2026).

### 2.6. Machine Learning and Gene-Phenotype Association Analysis

The random forest algorithm was used to evaluate the core genes in each target module and to rank the importance of gene features associated with lactation phenotypes. Because the lactation phenotypes were continuous variables, random forest regression models were implemented using the randomForest package (https://cran.r-project.org/package=randomForest; accessed on 10 February 2026) in R. The number of trees was set to 500 (ntree = 500), and the number of variables randomly selected at each split was set to the default value for regression models (mtry = p/3, where p is the number of input genes). Variable importance was calculated using importance = TRUE, and genes were ranked primarily based on the percentage increase in mean squared error (%IncMSE). To prioritize key candidate genes, a predefined threshold of relative contribution > 10% was applied. The relative contribution of each gene was calculated as (%IncMSE of a given gene/sum of %IncMSE values for all genes in the corresponding module) × 100%, based on the random forest model for each module. Pearson correlation analysis was then performed between candidate genes and each lactation phenotype, and genes significantly associated with multiple lactation traits were prioritized. Finally, candidate genes for subsequent analysis were identified based on random forest feature importance, Pearson correlation analysis, and published evidence.

### 2.7. Single-Gene GSEA of Candidate Genes

Using each candidate gene as the focal gene, Pearson correlation coefficients were calculated between its expression level and those of all other expressed genes, and all genes were ranked according to their correlation coefficients. Subsequently, GSEA was performed on the ranked gene lists using the gseGO and gseKEGG functions in the clusterProfiler R package (https://bioconductor.org/packages/clusterProfiler/; accessed on 12 February 2026) in R software. GO annotations were obtained from the Gene Ontology database, and KEGG pathway annotations were obtained from the Kyoto Encyclopedia of Genes and Genomes database. Together with the aforementioned module-level functional enrichment results, these analyses were used to systematically elucidate the potential biological functions of candidate genes in the regulation of lactation traits in dairy cows.

### 2.8. Analysis of Protein Interaction Networks of Candidate Genes

Candidate genes significantly associated with the same lactation phenotype in each module were submitted to the STRING database to construct a PPI network. The minimum required interaction score was set to 0.7, and the maximum number of displayed interactors was set to 20 [31]. The resulting PPI network was used to investigate potential interaction relationships among the candidate genes.

### 2.9. ROC Curve Plotting and Key Candidate Gene Screening

In this study, the phenotypic values of MFP, FPP, and TMS were divided into high- and low-value groups. ROC curve analysis was performed for candidate genes associated with these three phenotypes using the ROCit package (https://cran.r-project.org/package=ROCit; accessed on 20 February 2026) in R. The area under the ROC curve (AUC) was calculated to provide an exploratory assessment of the ability of each candidate gene to distinguish between the high- and low-value lactation phenotype groups within the present dataset. Because the same dataset was used for candidate gene discovery and ROC analysis, the AUC values were interpreted only as supportive evidence for candidate gene prioritization, rather than as an independent estimate of predictive performance. Finally, key candidate genes potentially involved in the regulation of lactation traits in dairy cows were prioritized by integrating the results of GSEA enrichment analysis, PPI network analysis, and ROC curve analysis.

### 2.10. RT-qPCR Validation

Three Holstein cows in their second parity at the middle to late lactation stage were selected for tissue collection. Lung, spleen, liver, heart, ovary, uterus, and mammary gland tissues were collected from each cow. After dissection, the tissue samples were immediately frozen in liquid nitrogen and transported to the laboratory for total RNA extraction and cDNA synthesis. RT-qPCR was performed to detect the expression levels of key candidate genes in different tissues.

Total RNA was extracted from tissue samples using the TRIzol method, and cDNA was synthesized using the ToloScript All-in-one RT EasyMix for qPCR kit (TOLOBIO, Shanghai, China). RT-qPCR was performed with three technical replicates on a Gentier 96R fully automatic medical PCR analysis system (Xi’an Tianlong Technology Co., Ltd., Xi’an, China) using the SYBR Green Premix Pro Taq HS qPCR Kit (Accurate Biotechnology, Changsha, China). The amplification program was as follows: initial denaturation at 95 °C for 30 s, followed by 40 cycles of denaturation at 95 °C for 5 s and annealing for 30 s. RT-qPCR primers were designed using Primer Premier 5.0. Primer sequences and annealing temperatures are listed in Table 3.

### 2.11. Statistical Analysis

All analyses and visualizations in this study were performed using R software version 4.4.1 (https://www.r-project.org/; accessed on 20 February 2026). Differences between two groups were analyzed using either Student’s *t*-test or a non-parametric test, depending on the distribution of the data. A nominal *p* < 0.05 was considered statistically significant for single-comparison analyses. For functional enrichment analyses, multiple testing correction was performed using the Benjamini–Hochberg false discovery rate (FDR) method, and adjusted *p* < 0.05 was considered statistically significant. For WGCNA module-trait correlation analysis, nominal *p* values were reported to identify phenotype-associated modules, and these results were interpreted as exploratory and were further prioritized using module membership, gene significance, PPI network analysis, GSEA, ROC analysis, and RT-qPCR validation. Relative expression levels of key candidate genes detected by RT-qPCR were calculated using the 2^−ΔΔCt^ method, with glyceraldehyde-3-phosphate dehydrogenase (*GAPDH*) used as the internal control for normalization.

## 3. Results

### 3.1. Screening of Hub Genes and Core Candidate Genes Through WGCNA

**Figure 1:**
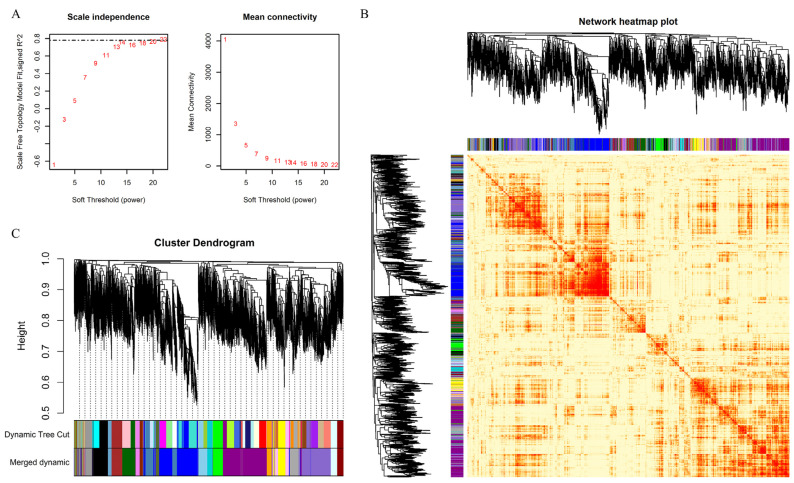
Construction of the weighted gene co-expression network. (**A**) Selection of the soft-thresholding power. The left panel shows the scale-free topology model fit, R^2^, across different soft-thresholding powers, while the right panel shows the corresponding mean connectivity; (**B**) The TOM heatmap of the co-expression network. Darker colors indicate higher topological overlap between genes; rows and columns represent individual genes; (**C**) Gene clustering dendrogram and module assignment. Each color represents a co-expression module. The first color bar indicates the original modules before merging, and the second color bar indicates the modules after merging.

After quality control and outlier removal, 10,568 genes from the transcriptome expression profiles were retained for WGCNA. A soft-thresholding power of 14 was selected based on a scale-free topology fit index threshold of R^2^ = 0.78. The TOM was then constructed, and co-expression modules were identified using hierarchical clustering with the dynamic tree cut algorithm. This analysis initially identified 43 co-expression modules, which were further merged according to module eigengene similarity greater than 0.75 (Supplementary Figure S1), resulting in 22 independent co-expression modules (Figure 1).

**Figure 2:**
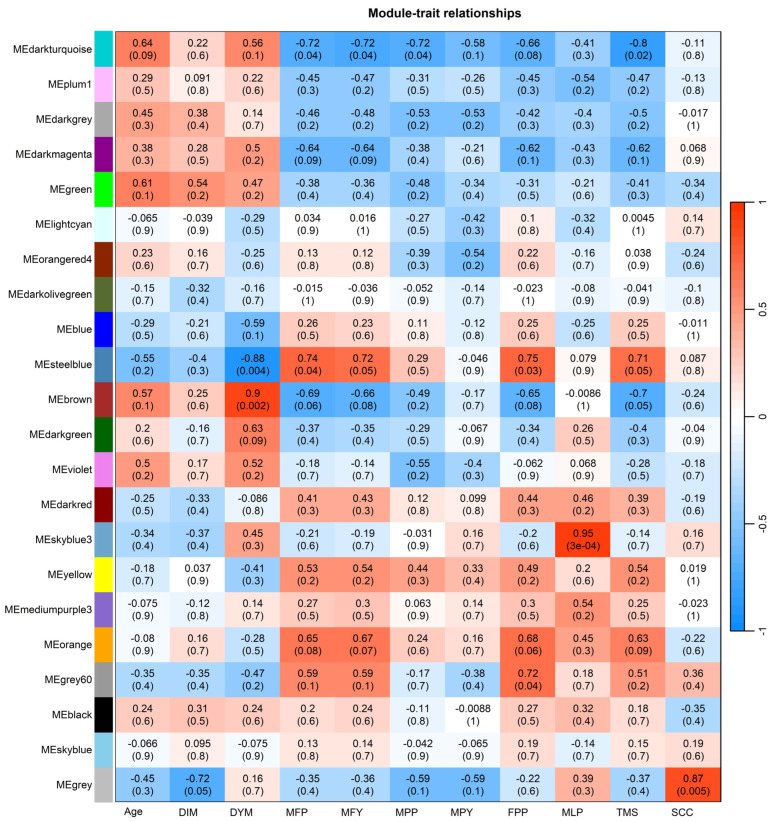
Module-phenotype correlation diagram. Each row represents a WGCNA co-expression module, and each column represents a lactation-related phenotype. The values in each cell represent the Pearson correlation coefficients. Red indicates positive correlations, whereas blue indicates negative correlations; the intensity of the color reflects the strength of the correlation. The colored labels on the left indicate the module colors assigned by WGCNA.

To identify modules associated with lactation traits, correlations between module eigengenes and lactation-related phenotypes were calculated, and a module-trait correlation matrix was constructed (Figure 2). The MEdarkturquoise module showed significant negative correlations with MFP, MFY, MPP, and TMS (*p* < 0.05). The MEsteelblue module was negatively correlated with DYM but positively correlated with MFP and FPP (*p* < 0.05). In contrast, the MEbrown and MEskyblue3 modules were positively correlated with DYM and MLP, respectively (*p* < 0.05). Based on these nominal associations, these four modules were selected as lactation trait-associated modules for subsequent exploratory analyses.

**Figure 3:**
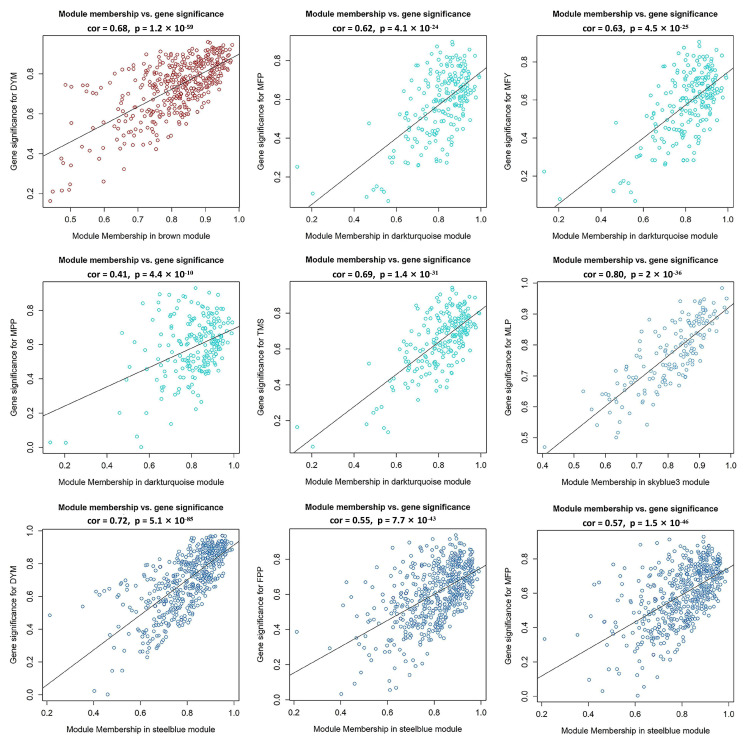
Scatter plots showing the relationship between GS and MM for genes in each module. Each dot represents one gene in the corresponding panel. The circles represent individual genes, and the lines represent the fitted linear regression lines.

The relationships between module genes and phenotypes were further evaluated by generating scatter plots of GS versus MM for each core module (Figure 3), with GS distribution visualized using histograms (Supplementary Figure S2). Significant positive correlations between GS and MM were detected in all four modules, further supporting their association with the corresponding phenotypes. Based on the criteria of GS ≥ 0.4 and MM ≥ 0.9, 55, 122, 106, and 31 hub genes were identified in the MEdarkturquoise, MEsteelblue, MEbrown, and MEskyblue3 modules, respectively. Furthermore, intramodular connectivity was calculated using the TOM, and the top 50 genes with the highest connectivity in each module were selected as core candidate genes.

### 3.2. Identification of Core Genes

**Figure 4:**
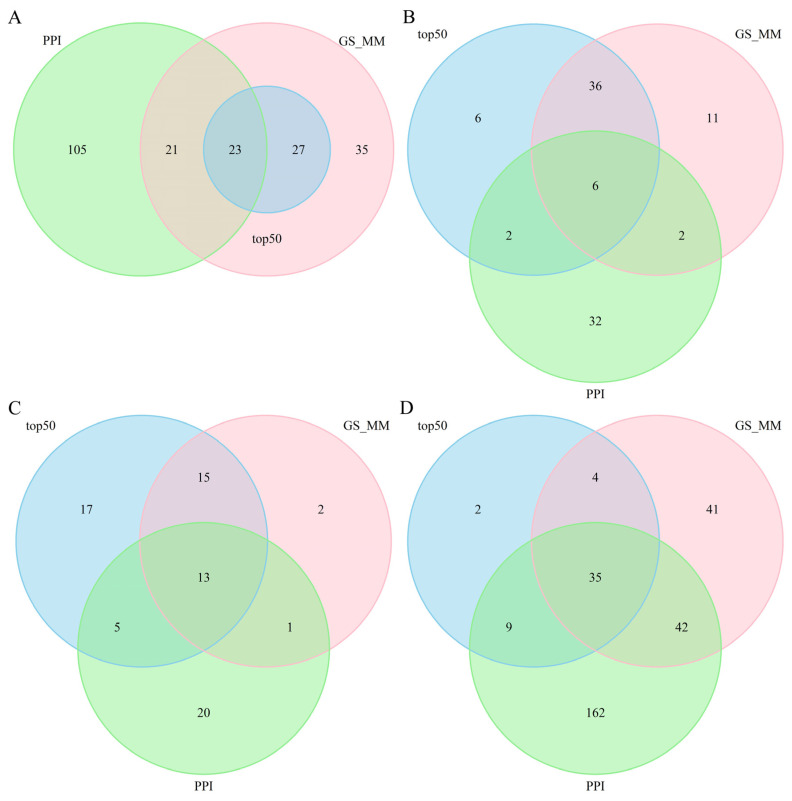
Venn diagrams of gene intersections for core gene identification in different modules. (**A**) MEbrown, 23 core genes; (**B**) MEdarkturquoise, 6 core genes; (**C**) MEskyblue3, 13 core genes; (**D**) MEsteelblue, 35 core genes.

PPI networks were constructed using the STRING database for genes in the MEdarkturquoise, MEsteelblue, MEbrown, and MEskyblue3 modules, which contained 215, 525, 430, and 158 genes, respectively. The resulting networks were imported into Cytoscape, and LAC values were calculated for each gene. Genes within the top 50% of LAC values were retained and then intersected with the GS-MM hub genes and the top 50 genes ranked by intramodular connectivity from WGCNA. As a result, 6, 35, 23, and 13 core genes were identified in the MEdarkturquoise, MEsteelblue, MEbrown, and MEskyblue3 modules, respectively (Figure 4).

### 3.3. Functional Enrichment Analysis of Core Genes

KEGG pathway and GO functional enrichment analyses were performed for the core genes in four modules significantly associated with lactation traits, with an adjusted *p* value < 0.05 used as the threshold for significant enrichment. The enrichment results are shown in Supplementary Figure S3, and the major enriched pathways of the core genes are presented in Table 4. Therefore, only the main functional patterns are summarized here to avoid duplication between the text and the figures/tables.

Overall, the enrichment results indicated that the core genes in the lactation-related modules were mainly involved in protein synthesis and degradation, mitochondrial energy metabolism, oxidative stress regulation, immune response, and cell cycle-related biological processes. In the MEbrown module, the enriched pathways and GO terms were primarily associated with ribosome-related translation, ubiquitin-mediated protein degradation, and signaling pathways related to cellular metabolism and lactation regulation. The MEskyblue3 module was mainly enriched in immune- and inflammation-related processes, extracellular matrix organization, and mitochondrial or cytosolic cellular components. For the MEdarkturquoise module, enrichment patterns were highly consistent across the four milk component phenotypes, supporting the reliability of the enrichment results. The core genes in this module were mainly associated with ribosome function, oxidative phosphorylation, glutathione metabolism, peroxisome-related processes, and oxidation–reduction regulation. In the MEsteelblue module, enrichment results differed among associated phenotypes but were mainly related to DNA damage repair, cell cycle regulation, cellular senescence, ubiquitin-mediated proteolysis, fatty acid biosynthesis, and mitochondrial energy metabolism. Taken together, the functional enrichment analyses revealed biologically coherent patterns across the target modules and highlighted several key processes closely related to lactation traits, including translation, protein degradation, mitochondrial function, oxidative phosphorylation, immune response, lipid metabolism, and cell cycle regulation.

### 3.4. Machine Learning and Correlation Analysis

**Figure 5:**
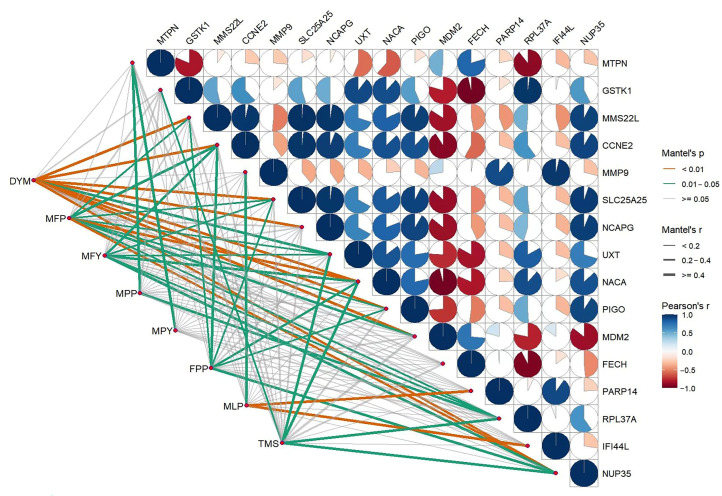
Correlation analysis between 16 genes and lactation phenotypes. Line thickness represents the strength of the correlation, and darker colors indicate higher statistical significance. The sector size within each circle represents the strength of the correlation; larger sectors indicate higher absolute Pearson’s r values.

A lactation phenotype prediction model was constructed using the core genes of each module based on the random forest algorithm. The genes were ranked according to their feature importance scores (Supplementary Figure S4), and phenotypic association analysis was performed for the selected genes (Figure 5).

In the MEbrown module, the random forest model identified *RPS*26, *ATP6V0A*1, *NACA*, *SETD*7, *MDM*2, and *UXT* as the top six contributors to model prediction, with each gene contributing more than 10%. Among them, *NACA*, *MDM*2, and *UXT* were repeatedly involved in enriched terms related to DYM in the previous enrichment analysis. In the MEskyblue3 module, *PARP*14 and *IFI44L* showed the highest feature importance scores, with individual contributions exceeding 15%. In the MEdarkturquoise module, *RPL37A* and *GSTK*1 ranked highly in the prediction models for four milk component-related phenotypes, with individual contributions exceeding 19%. Specifically, *RPL37A* ranked first in the MPP, MFY, and TMS models, whereas *GSTK*1 ranked first in the MFP model. In the MEsteelblue module, *PIGO* was associated with milk fat-related traits and showed high feature importance in the MFP prediction model, with an individual contribution exceeding 10%. *NUP*35 showed an individual contribution of more than 30% in the DYM prediction model. In addition, based on the integrated enrichment results and previous reports, *MMS22L* and *NCAPG* were suggested to be associated with FPP, whereas *CCNE*2 and *SLC25A*25 were potentially associated with MFP. Further association analysis between genes with high trait contributions and lactation phenotypes showed that *MMS22L*, *CCNE*2, *SLC25A*25, *UXT*, *NACA*, *PIGO*, *MDM*2, *RPL37A*, and *NUP*35 were significantly correlated with multiple lactation traits (*p* < 0.05). Therefore, these nine genes were selected as candidate genes for subsequent analyses.

### 3.5. Single-Gene GSEA

Single-gene GSEA was performed for the nine candidate genes identified above. The results shown in Supplementary Figure S5 indicated that the GSEA results for *UXT* and *PIGO* did not overlap with the GO terms or KEGG pathways identified by KOBAS enrichment analysis; therefore, these two genes were excluded from subsequent analysis. The remaining seven genes showed significant positive enrichment peaks (*p* < 0.05), with peak values ranging from 0.2 to 0.6. Specifically, *MDM*2 was significantly enriched in two GO terms, “positive regulation of proteasomal ubiquitin-dependent protein catabolic process” and “ubiquitin protein ligase activity”, as well as in three KEGG pathways, “Endocrine resistance”, “FoxO signaling pathway”, and “PI3K-Akt signaling pathway”. *NACA* was significantly enriched in the GO term “protein targeting to membrane”. *RPL37A* was significantly enriched in three GO terms, “structural constituent of ribosome”, “ribosome”, and “translation”, as well as in the KEGG pathway “Ribosome”. *NUP*35 was significantly enriched in two GO terms, “nucleoplasm” and “identical protein binding”. *CCNE*2 was significantly enriched in two GO terms, “G1/S transition of mitotic cell cycle” and “mitotic cell cycle phase transition”, as well as in the KEGG pathway “Cell cycle”. *MMS22L* was significantly enriched in the GO term “double-strand break repair via homologous recombination”. *SLC25A*25 was significantly enriched in two GO terms, “cellular respiration” and “ATP metabolic process”.

### 3.6. PPI Analysis of Candidate Genes

**Figure 6:**
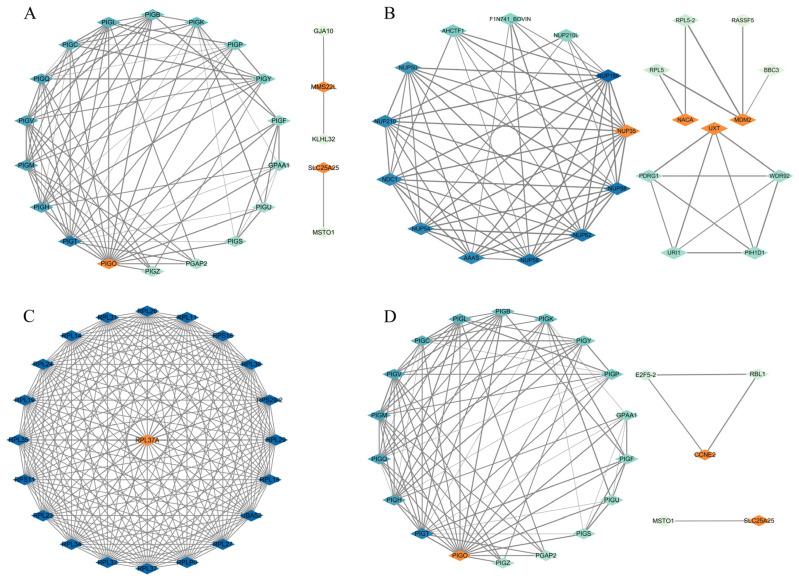
Protein–protein interaction networks of candidate genes and their associated proteins. Orange nodes indicate proteins encoded by candidate genes, while blue nodes indicate associated interacting proteins. Darker blue represents a stronger association. (**A**) FPP-associated genes: *PIGO*, *SLC25A*25, and *MMS22L*; (**B**) DYM-associated genes: *NUP*35, *NACA*, *UXT*, and *MDM*2. (**C**) TMS-associated gene: *RPL37A*; (**D**) MFP-associated genes: *PIGO*, *CCNE*2, and *SLC25A*25.

A PPI network of the nine candidate genes was constructed using the STRING database with a minimum confidence score of 0.7 and a maximum number of interactors of 20 (Figure 6). Among the candidate genes associated with FPP, PIGO, CCNE2, MMS22L, and SLC25A25 were included in the network. No high-confidence interacting proteins were predicted for CCNE2. Most proteins interacting with PIGO belonged to the PIG family, which is mainly involved in the glycosylphosphatidylinositol (GPI) biosynthesis pathway. MSTO1, an interacting protein of SLC25A25, is a mitochondrial functional protein. Among the candidate genes associated with DYM, NUP35, NACA, MDM2, and UXT were included in the PPI network. NACA and MDM2 showed indirect interactions through RPL5/RPL5-2, and RPL family proteins are core structural components of the large ribosomal subunit. NUP35 mainly interacted with members of the nuclear pore complex (NUP) family. Among the candidate genes associated with MFP, PIGO, CCNE2, and SLC25A25 were included in the network. The interaction patterns of PIGO and SLC25A25 were largely consistent with those observed for FPP. The interacting proteins RBL1 and E2F5-2 associated with CCNE2 are involved in cell cycle progression and transcriptional regulation. RPL37A was the only candidate gene associated with TMS, and all of its interacting proteins belonged to the RPL family.

### 3.7. ROC Curves and Screening of Key Candidate Genes

**Figure 7:**
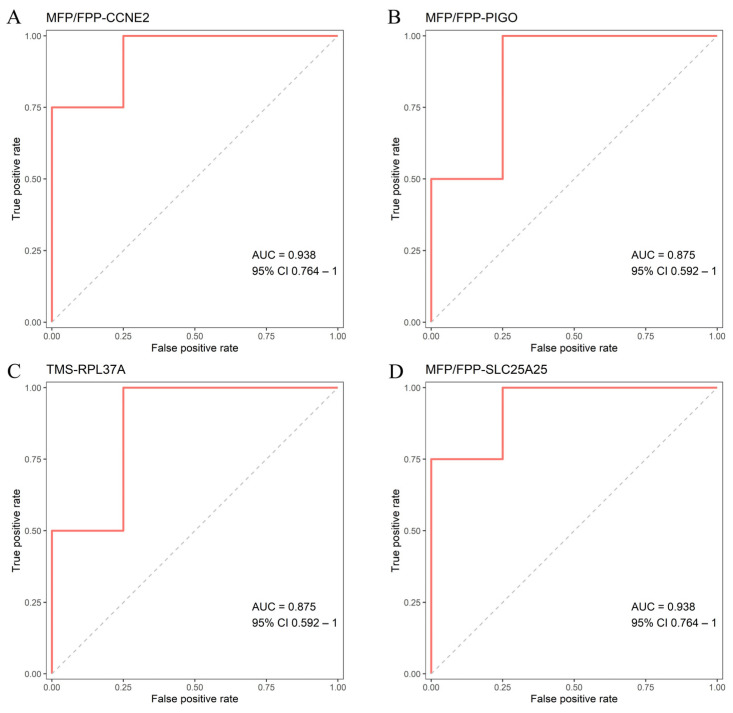
ROC curves of candidate genes. The red solid line represents the ROC curve, and the gray dashed line represents the reference line for random classification. The area under the curve (AUC) and its 95% confidence interval (CI) are displayed within each panel. A larger AUC indicates better discriminatory ability. (A) ROC curve of CCNE2 for MFP and FPP. (B) ROC curve of PIGO for MFP and FPP. (C) ROC curve of RPL37A for TMS. (D) ROC curve of SLC25A25 for MFP and FPP.

ROC analysis was performed for the candidate genes associated with the MFP, FPP, and TMS phenotypes (Figure 7). The results showed that *CCNE*2 (AUC = 0.938, 95% CI: 0.764–1.000) and *SLC25A*25 (AUC = 0.938, 95% CI: 0.764–1.000) exhibited higher AUC values than *PIGO* (AUC = 0.875, 95% CI: 0.592–1.000) in distinguishing between the high- and low-MFP groups. In addition, *RPL37A* showed moderately high discriminatory performance for distinguishing between the high- and low-TMS groups (AUC = 0.875, 95% CI: 0.592–1.000). By integrating the results of GSEA enrichment analysis, PPI network analysis, and ROC curve analysis, four key candidate genes, namely *RPL37A*, *SLC25A*25, *CCNE*2, and *MDM*2, were ultimately identified as potential regulators of bovine lactation traits. Specifically, *RPL37A* may be involved in the regulation of MFP and TMS in dairy cows, *MDM*2 may contribute to the regulation of DYM, and *SLC25A*25 and *CCNE*2 may jointly participate in the regulation of MFP.

### 3.8. RT-qPCR Validation of Key Candidate Genes

**Figure 8:**
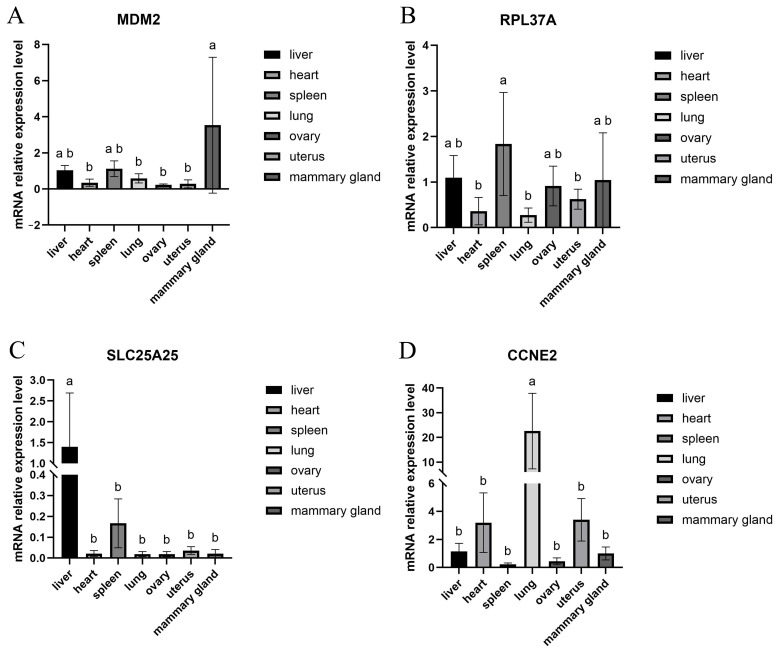
Relative expression levels of key candidate genes associated with lactation traits in different tissues. (**A**–**D**) Expression levels of *MDM*2, *RPL37A*, *SLC25A*25, and *CCNE*2, respectively. Different lowercase letters indicate significant differences among tissues (*p* < 0.05).

To examine the tissue expression patterns of *MDM*2, *RPL37*A, *SLC25A*25, and *CCNE*2, their relative mRNA levels were measured by RT-qPCR across seven tissues, including mammary gland, heart, liver, spleen, lung, ovary, and uterus (Figure 8). The results showed that *MDM*2 expression was significantly higher in the mammary gland than in heart, lung, ovary, and uterus (*p* < 0.05). *RPL37*A was widely expressed across multiple tissues, with particularly high expression in spleen, which was significantly higher than that in heart, lung, and uterus (*p* < 0.05). Although its expression in the mammary gland, liver, and ovary did not differ significantly from that in spleen, *RPL37*A also showed relatively high expression levels in these tissues. *SLC25A*25 exhibited liver-enriched expression, with significantly higher relative mRNA levels in liver than in other tissues (*p* < 0.05). *CCNE*2 showed the highest expression in lung tissue (*p* < 0.05), moderate expression in heart and uterus, and low expression in liver, spleen, ovary, and mammary gland. These RT-qPCR results confirmed the tissue expression patterns of the four candidate genes and provided preliminary experimental support for their biological relevance. However, these results do not directly validate the computational associations between these genes and lactation phenotypes, which require further confirmation in independent populations and functional studies.

## 4. Discussion

In the present study, WGCNA identified four co-expression modules, namely MEbrown, MEskyblue3, MEdarkturquoise, and MEsteelblue, that were significantly associated with lactation traits. Functional enrichment analysis showed that genes within these modules were mainly enriched in pathways related to ribosome function, the cell cycle, oxidative phosphorylation, and PI3K-Akt signaling. In particular, the PI3K-Akt signaling pathway is a classical regulatory pathway involved in BMECs proliferation and milk fat synthesis [32,33]. By integrating WGCNA, PPI network analysis, GO and KEGG enrichment analyses, random forest analysis, and multi-step bioinformatics screening, four key candidate genes, namely *MDM*2, *RPL37A*, *SLC25A*25, and *CCNE*2, were identified from the lactation-related modules. These findings are consistent with our hypothesis that lactation traits may be associated with specific co-expression mod-ules and candidate genes involved in mammary epithelial cell activity, energy metabolism, and milk component synthesis, thereby helping to achieve the objective of identifying potential molecular candidates and biological pathways related to lactation performance in dairy cattle.

As a member of the E3 ubiquitin ligase family, *MDM*2 has previously been reported to be associated with milk yield-related traits in Chinese Holstein cows [34,35]. In the present study, single-gene GSEA suggested that *MDM*2 was potentially related to ubiquitin-mediated proteolysis and cell cycle-associated pathways, implying a possible role in BMECs proliferation. RT-qPCR results showed relatively higher expression of *MDM*2 in mammary tissue, which may provide preliminary evidence for its relevance to mammary gland function and DYM. Considering the close relationship between BMECs proliferation, cellular activity, and milk secretion, *MDM*2 might be involved in lactation-related biological processes, possibly through effects on cell cycle progression and protein turnover. In addition, PPI analysis suggested a potential connection between *MDM*2 and RPL family proteins [36,37], indicating that *MDM*2 may be indirectly associated with ribosome-related processes and functionally related to *RPL37A* in BMECs proliferation or protein synthesis. However, further experimental validation is required to clarify the precise regulatory role of *MDM*2 in mammary epithelial cells.

*RPL37A* encodes a structural component of the 60S large ribosomal subunit and is involved in ribosome assembly and protein translation [38,39]. In this study, the relatively high expression of *RPL37A* in mammary tissue suggests that it may be associated with protein synthesis activity in BMECs. Previous studies have reported that ribosomal protein family genes are related to milk protein biosynthesis in BMECs [40,41], supporting the potential relevance of *RPL37A* to lactation-related processes. Moreover, proteins connected with *RPL37A* in the PPI network were mainly members of the RPL family, suggesting that *RPL37A* may be associated with ribosome-related biological functions. Therefore, *RPL37A* may represent a candidate gene related to milk protein synthesis and TMS.

Milk fat synthesis is closely linked to cellular energy metabolism. *SLC25A*25 belongs to the SLC25A mitochondrial transporter family, whose members are generally involved in mitochondrial substrate transport and ATP metabolism [42,43,44,45]. In the present study, *SLC25A*25 showed higher expression in liver than in the other examined tissues, suggesting a possible association with hepatic mitochondrial energy metabolism. Although its direct involvement in mammary tissue remains unclear, *SLC25A*25 may be indirectly related to milk fat traits through systemic energy metabolism or hepatic lipid metabolism. In addition, PPI analysis suggested a potential connection between *SLC25A*25 and *MSTO*1, a protein associated with mitochondrial function [46,47], further indicating that *SLC25A*25 may be linked to mitochondrial homeostasis. These findings suggest that *SLC25A*25 is a candidate gene potentially associated with MFP, but further functional studies are needed to confirm this possibility.

*CCNE2* is known as a regulator of the G1/S phase transition and has been implicated in cell proliferation and differentiation [48,49,50,51]. Although RT-qPCR analysis showed relatively low expression of *CCNE*2 in mammary tissue, its association with cell cycle-related processes may still be relevant to lactation biology. Low expression in mammary tissue does not necessarily exclude a potential role in specific cell populations, developmental stages, or physiological conditions. Given the importance of BMECs proliferation and differentiation for mammary gland function, *CCNE*2 may be associated with lactation-related traits through cell cycle-related pathways. Furthermore, the presence of *RBL*1 and E2F family members in the *CCNE*2-centered PPI network [52,53,54,55,56] suggests a possible link between *CCNE*2, cell cycle progression, and transcriptional regulation. Nevertheless, additional experimental validation is required to determine whether *CCNE*2 has a direct functional role in lactation.

However, this study has some limitations. The candidate genes identified here were mainly screened based on transcriptomic data and bioinformatics analyses, and their functional roles in lactation traits require further experimental validation. In addition, although the present dataset provided useful information for identifying lactation-related modules and candidate genes, further studies using larger independent populations would help improve the robustness and generalizability of these findings. Therefore, in vitro or in vivo functional experiments are needed to validate the potential roles of these candidate genes and to further clarify the molecular mechanisms underlying lactation performance in dairy cattle.

## 5. Conclusions

This study integrated WGCNA, bioinformatics analysis, machine learning-based prioritization, and RT-qPCR tissue expression analysis to identify potential candidate genes associated with lactation-related traits in Holstein cows. Specifically, *MDM*2 was associated with DYM, *RPL37A* with TMS, and *SLC25A*25 and *CCNE*2 with MFP in the computational analyses. Functional enrichment results suggested that these genes may be involved in biological processes related to milk component synthesis, mitochondrial energy metabolism, and cell cycle-related pathways. RT-qPCR analysis confirmed the tissue expression patterns of these candidate genes, providing preliminary support for their biological relevance. Overall, these findings contribute to the identification of lactation-related co-expression modules and highlight *MDM*2, *RPL37A*, *SLC25A*25, and *CCNE*2 as promising candidate genes for future functional studies. Further validation in larger independent populations and mechanistic investigations using in vitro and in vivo experiments are required to clarify their potential roles in lactation traits and to evaluate their possible application in dairy cattle molecular breeding programs.

## Figures and Tables

**Table 1:** Ingredient and nutrient composition in diet (dry matter basis) %.

Ingredient	Content	Composition	Content
Alfalfa	16.22	Dry matter (kg)	17.28
Corn silage	51.32	Net energy for lactating cow (MJ/kg)	7.76
Tablet corn	10.82	Crude protein	18.31
Soybean meal	10.82	Neutral detergent fiber	35.84
Cotton meal	5.41	Acid detergent fiber	21.85
10% premix	5.41	Fat	2.59
		Ca	0.52
Total	100	P	0.35

Note: Each kg of premix contains 800,000 IU of VA, 200,000 IU of VD, 4000 mg of VE, 1200 mg of Cu, 6000 mg of Fe, 4000 mg of Mn, 4000 mg of Zn, 40 mg of I, 40 mg of Co and 32 mg of Se.

**Table 2:** Phenotypic data of lactation traits in Holstein cows.

Items	ID	Age (Month)	DIM (Day)	DYM (kg)	MFP (%)	MFY (kg)	MPP (%)	MPY (kg)	FPP	MLP (%)	TMS (kg)	SCC (10,000 per mL)
HMF	H_2098	30	186	35.65	4.82	1.72	3.78	1.35	1.28	5.06	14.02	5
	H_2046	31	189	36.54	4.54	1.66	3.59	1.31	1.26	5.11	13.64	2
	H_2226	29	160	35.66	4.74	1.69	3.36	1.20	1.41	5.13	13.64	9
	H_2190	29	157	35.21	4.88	1.72	3.76	1.32	1.30	5.03	14.12	5
LMF	L_2170	30	207	36.74	2.85	1.05	3.55	1.30	0.80	4.95	11.98	7
	L_2034	31	187	36.56	2.6	0.95	3.21	1.17	0.81	5.01	11.28	6
	L_2037	31	175	37.25	2.81	1.05	3.36	1.25	0.84	4.97	11.53	5
	L_2137	29	150	37.21	2.84	1.06	3.58	1.33	0.79	5.38	12.09	7

Note: HMF, high milk fat; LMF, low milk fat; ID, identification number of each cow.

**Table 3:** Primer sequence and annealing temperature.

Gene	Primer Type	Primer Sequence (5′–3′)	Product Size/bp	Tm/°C
*RPL37A*	Forward	CAAGAAGGTCGGAATCGT	163	53.0
	Reverse	GGAACCACAGTGCCAAAT		
*SLC25A*25	Forward	GCCATCGCCCAGAGTAGT	150	55.0
	Reverse	GGGGACGTAGCCTTTGTAG		
*MDM*2	Forward	AGGGGATTTGTTTGGAGT	106	53.0
	Reverse	TGATGGTTCTGCTTGCTG		
*CCNE*2	Forward	TGAGTTGGAACCACAGATG	194	51.7
	Reverse	CAAGTTTGGAAGCAATGAA		
*GAPDH*	Forward	TCGGAGTGAACGGATTCGG	192	60.0
	Reverse	TGATGACGAGCTTCCCGTTC		

**Table 4:** Main functional enrichment results of the core genes.

Module	Phenotype	Gene	Database	Term	Adjusted *p* Value
MEbrown	DYM	*MDM*2	KEGG	PI3K-Akt signaling pathway	0.035
				FoxO signaling pathway	0.005
				Endocrine resistance	0.003
			GO	positive regulation of proteasomal ubiquitin-dependent protein catabolic process	0.012
				ubiquitin protein ligase activity	1.25 × 10^−5^
				regulation of transcription by RNA polymerase II	0.01
		*NACA*	GO	protein targeting to membrane	0.024
				positive regulation of nucleic acid-templated transcription	0.043
		*UXT*	GO	regulation of transcription by RNA polymerase II	0.010
MEskyblue3	MLP	*MMP*9	GO	extracellular matrix organization	0.001
		*PARP*14	GO	cytoplasm	0.033
				cytosol	0.021
MEdarkturquoise	MFP MFY MPP TMS	*RPL37A*	KEGG	Ribosome	0.033
			GO	metal ion binding	0.022
				structural constituent of ribosome	0.032
				ribosome	0.006
				translation	0.026
		*GSTK*1	KEGG	Glutathione metabolism	0.013
				Metabolic pathways	0.002
				Peroxisome	0.017
			GO	mitochondrion	0.010
				peroxisome	0.014
				protein disulfide oxidoreductase activity	0.004
				epithelial cell differentiation	0.010
				cellular oxidant detoxification	0.014
		*MTPN*	GO	regulation of cell size	0.004
				positive regulation of cell growth	0.011
		*FECH*	GO	mitochondrion	0.010
				mitochondrial inner membrane	0.047
MEsteelblue	DYM MFP FPP	*CCNE*2	KEGG	Cell cycle	0.0004
			GO	G1/S transition of mitotic cell cycle	0.041
				mitotic cell cycle phase transition	0.023
		*SLC25A*25	GO	cellular respiration	0.014
				ATP metabolic process	0.027
		*NUP*35	GO	nucleoplasm	1.10 × 10^−6^
				identical protein binding	4.78 × 10^−5^
		*MMS22L*	GO	double-strand break repair via homologous recombination	9.47 × 10^−7^
		*PIGO*	KEGG	Glycosylphosphatidylinositol (GPI)-anchor biosynthesis	0.032

## Data Availability

The original data presented in the study are openly available in SRA database with the accession number PRJNA730595. Access to the data of permanent link to https://www.ncbi.nlm.nih.gov/sra/PRJNA730595, accessed on 2 January 2026.

## Supplementary Materials

The following supporting information can be downloaded at: https://www.mdpi.com/article/10.3390/vetsci13070693/s1, Figure S1: Cluster tree clipping plot, below the red line are the modules with a similarity greater than 75%.; Figure S2: tHistogram of GS distribution of each trait. The representative traits of A-G were DYM, FPP, MFP, MFY, MLP, MPP, and TMS.; Figure S3: Functional enrichment analysis of core genes, the size of the bubble represents the number of enriched genes, the color of the bubble represents the significance, the larger the bubble, the more enriched genes of the pathway/item, the darker the color of the bubble, and the higher the significance. (A) Functional enrichment map of MEbrown core genes on DYM. (B) MLP functional enrichment diagram of MEskyblue3 core gene. (C-F) Functional enrichment diagram of MEdarkturquoise core genes for MFP, MFY, MPP, TMS. (G-I) Functional enrichment diagram of MEsteelblue core genes for DYM, FPP, MFP.; Figure S4: Machine-learning score plots for genes in trait-associated modules. (A) Machine-learning scores of genes in the MEbrown module for DYM. (B) Machine-learning scores of genes in the MEskyblue3 module for MLP. (C-F) Machine-learning scores of genes in the MEdarkturquoise module for MFP, MFY, MPP, and TMS, respectively. (G-I) Machine-learning scores of genes in the MEsteelblue module for DYM, FPP, and MFP, respectively.; Figure S5: Single-gene GSEA plots. The curves show the enrichment of GO terms and KEGG pathways associated with each gene, and the peak indicates the maximum enrichment score. (A,B) GO term and KEGG pathway enrichment plots for *CCNE*2. (C) GO term enrichment plot for *NUP*35. (D) GO term enrichment plot for *SLC25A*25. (E,F) GO term and KEGG pathway enrichment plots for *RPL37A*. (G) GO term enrichment plot for *MMS22L*. (H) GO term enrichment plot for *NACA*. (I,J) GO term and KEGG pathway enrichment plots for *MDM*2.

## Abbreviations

The following abbreviations are used in this manuscript:

WGCNA	Weighted gene co-expression network analysis
BMECs	Bovine mammary epithelial cells
RT-qPCR	Reverse transcription quantitative real-time PCR
GSEA	Gene set enrichment analysis
PPI	Protein–protein interaction
DIM	Days in milk
DYM	Daily milk yield
MFP	Milk fat percentage
MFY	Milk fat yield
MPP	Milk protein percentage
MPY	Milk protein yield
MLP	Milk lactose percentage
TMS	Total milk solids
FPP	Fat-to-protein percentage ratio
SCC	Somatic cell count
DHI	Dairy herd improvement
TOM	Topological overlap matrix
MM	Module membership
GS	Gene significance
