# Supplementary material for: Identification of Key Candidate Genes Potentially Associated with Lactation Traits in Dairy Cows Using Weighted Gene Co-Expression Network Analysis
Source: Vet Sci. 2026 Jul 16;13(7):693. doi: 10.3390/vetsci13070693 (PMC13431634; doi:10.3390/vetsci13070693)
Supplement: Supplementary file 1 [file vetsci-13-00693-s001.zip › vetsci-4423423-supplementary.pdf]

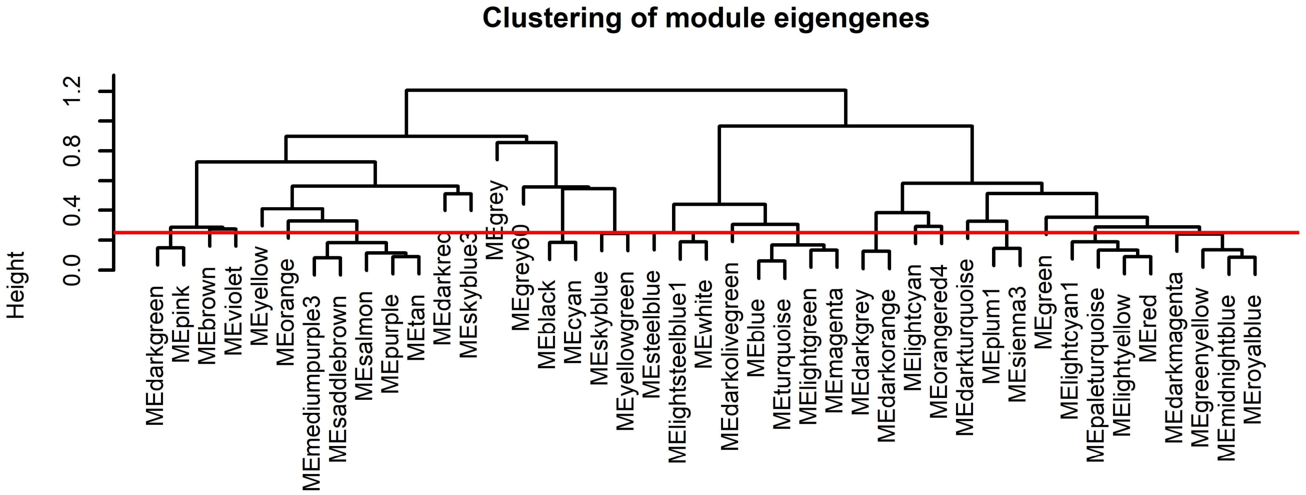

Supplementary Figure S1. Cluster tree clipping plot, below the red line are the modules with a similarity greater than 75%.

A

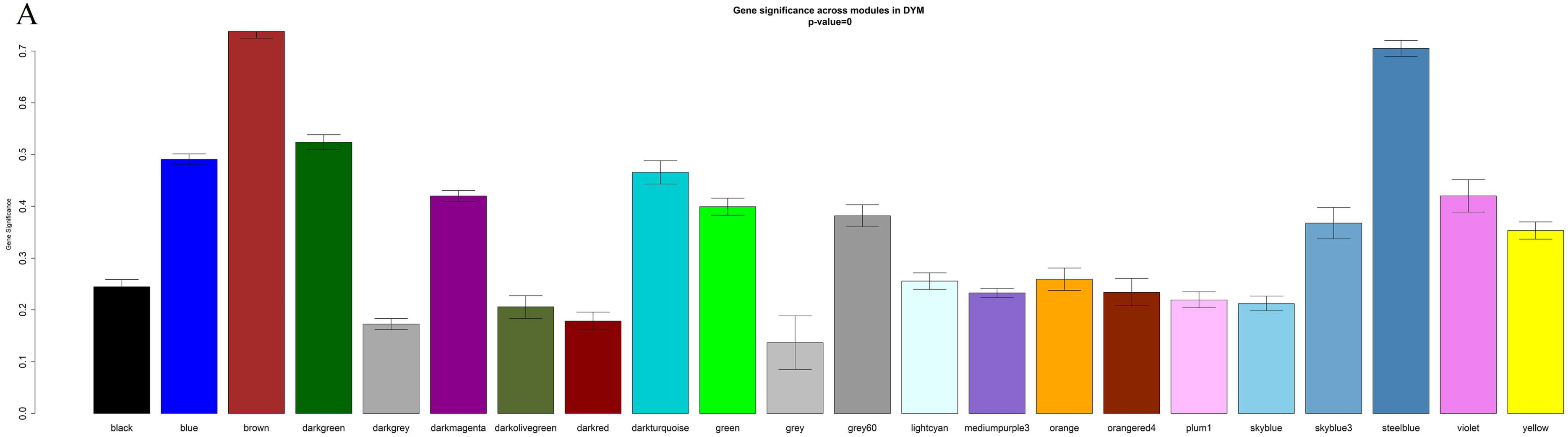
Gene significance across modules in DYM
p-value=0
Gene Significance
0.0
0.1
0.2
0.3
0.4
0.5
0.6
0.7
black
blue
brown
darkgreen
darkgrey
darkmagenta
darkolivegreen
darkred
darkturquoise
green
grey
grey60
lightcyan
mediumpurple3
orange
orangered4
plum1
skyblue
skyblue3
steelblue
violet
yellow

B

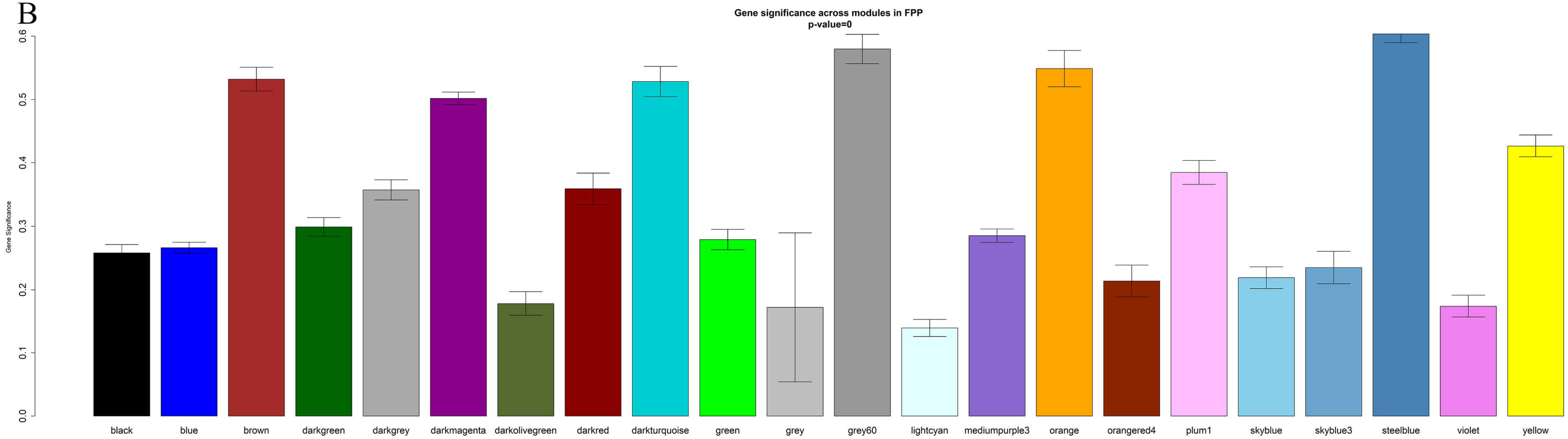
Gene significance across modules in FPP
p-value=0
Gene Significance
0.0
0.1
0.2
0.3
0.4
0.5
0.6
black
blue
brown
darkgreen
darkgrey
darkmagenta
darkolivegreen
darkred
darkturquoise
green
grey
grey60
lightcyan
mediumpurple3
orange
orangered4
plum1
skyblue
skyblue3
steelblue
violet
yellow

C

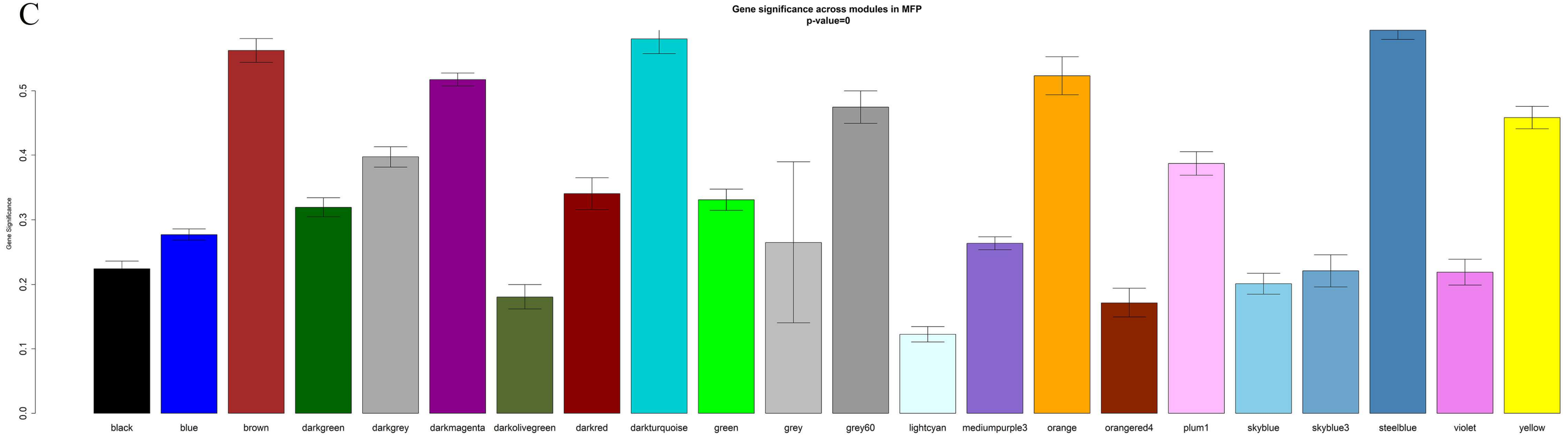
Gene significance across modules in MFP
p-value=0
Gene Significance
0.0
0.1
0.2
0.3
0.4
0.5
black
blue
brown
darkgreen
darkgrey
darkmagenta
darkolivegreen
darkred
darkturquoise
green
grey
grey60
lightcyan
mediumpurple3
orange
orangered4
plum1
skyblue
skyblue3
steelblue
violet
yellow

D

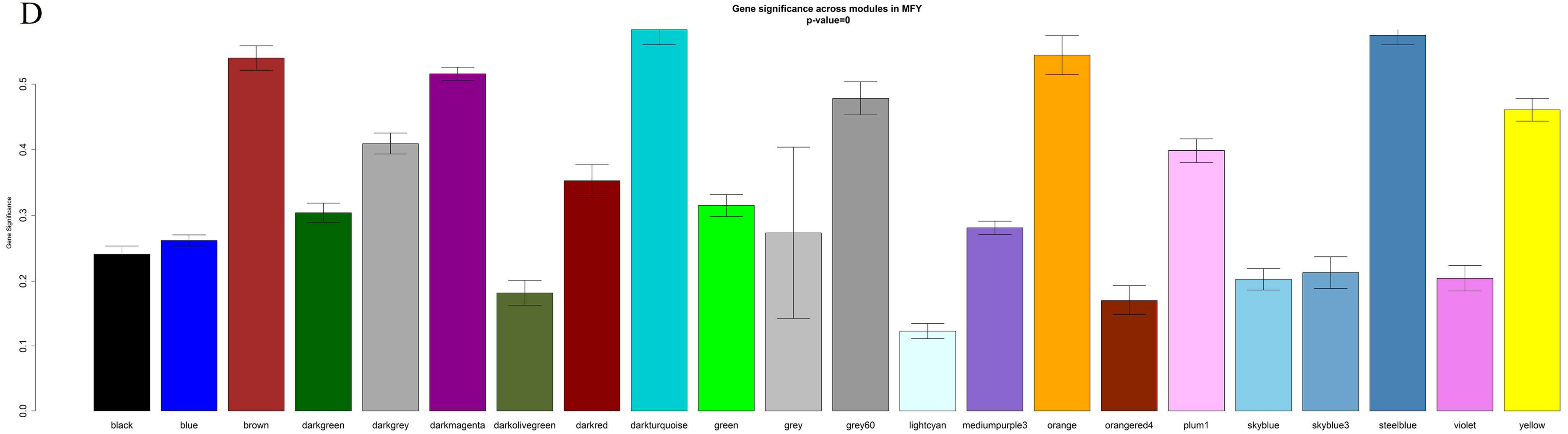
Gene significance across modules in MFY
p-value=0
Gene Significance
0.0
0.1
0.2
0.3
0.4
0.5
black
blue
brown
darkgreen
darkgrey
darkmagenta
darkolivegreen
darkred
darkturquoise
green
grey
grey60
lightcyan
mediumpurple3
orange
orangered4
plum1
skyblue
skyblue3
steelblue
violet
yellow

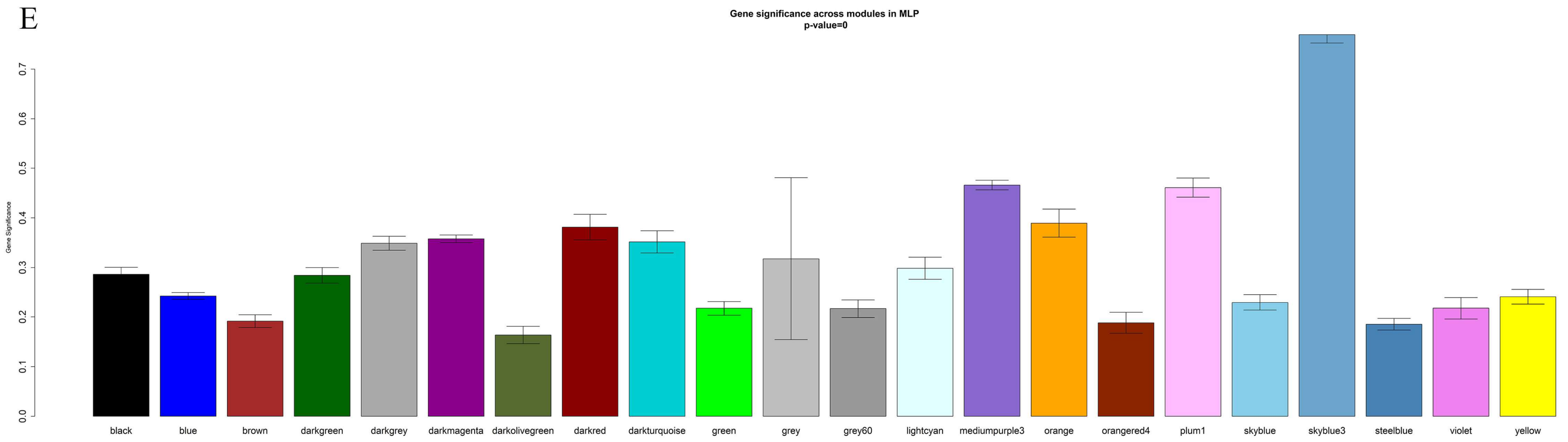
E
Gene significance across modules in MLP
p-value=0
Gene Significance
0.0
0.1
0.2
0.3
0.4
0.5
0.6
0.7
black
blue
brown
darkgreen
darkgrey
darkmagenta
darkolivegreen
darkred
darkturquoise
green
grey
grey60
lightcyan
mediumpurple3
orange
orangered4
plum1
skyblue
skyblue3
steelblue
violet
yellow

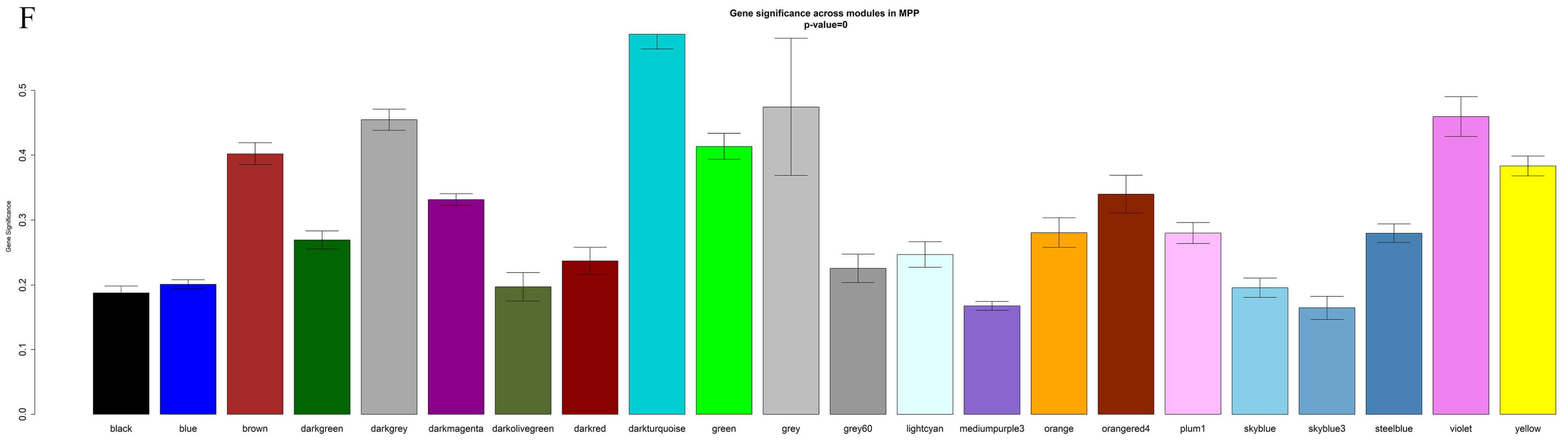
F
Gene significance across modules in MPP
p-value=0
Gene Significance
0.0
0.1
0.2
0.3
0.4
0.5
black
blue
brown
darkgreen
darkgrey
darkmagenta
darkolivegreen
darkred
darkturquoise
green
grey
grey60
lightcyan
mediumpurple3
orange
orangered4
plum1
skyblue
skyblue3
steelblue
violet
yellow

G

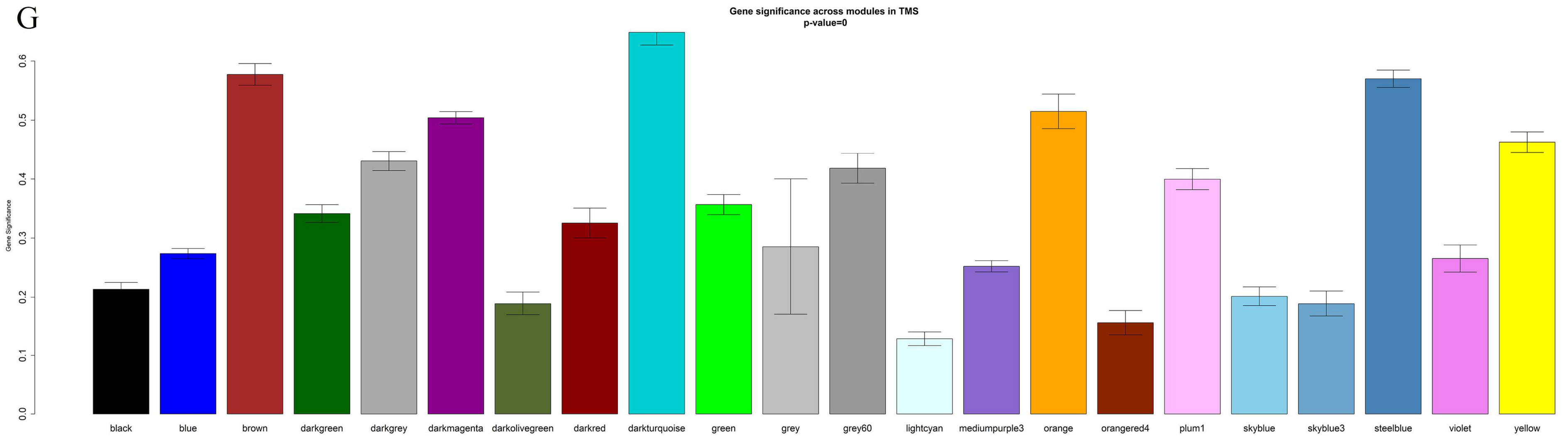

Supplementary Figure S2. Histogram of GS distribution of each trait. The representative traits of A-G were DYM, FPP, MFP, MFY, MLP, MPP, and TMS.

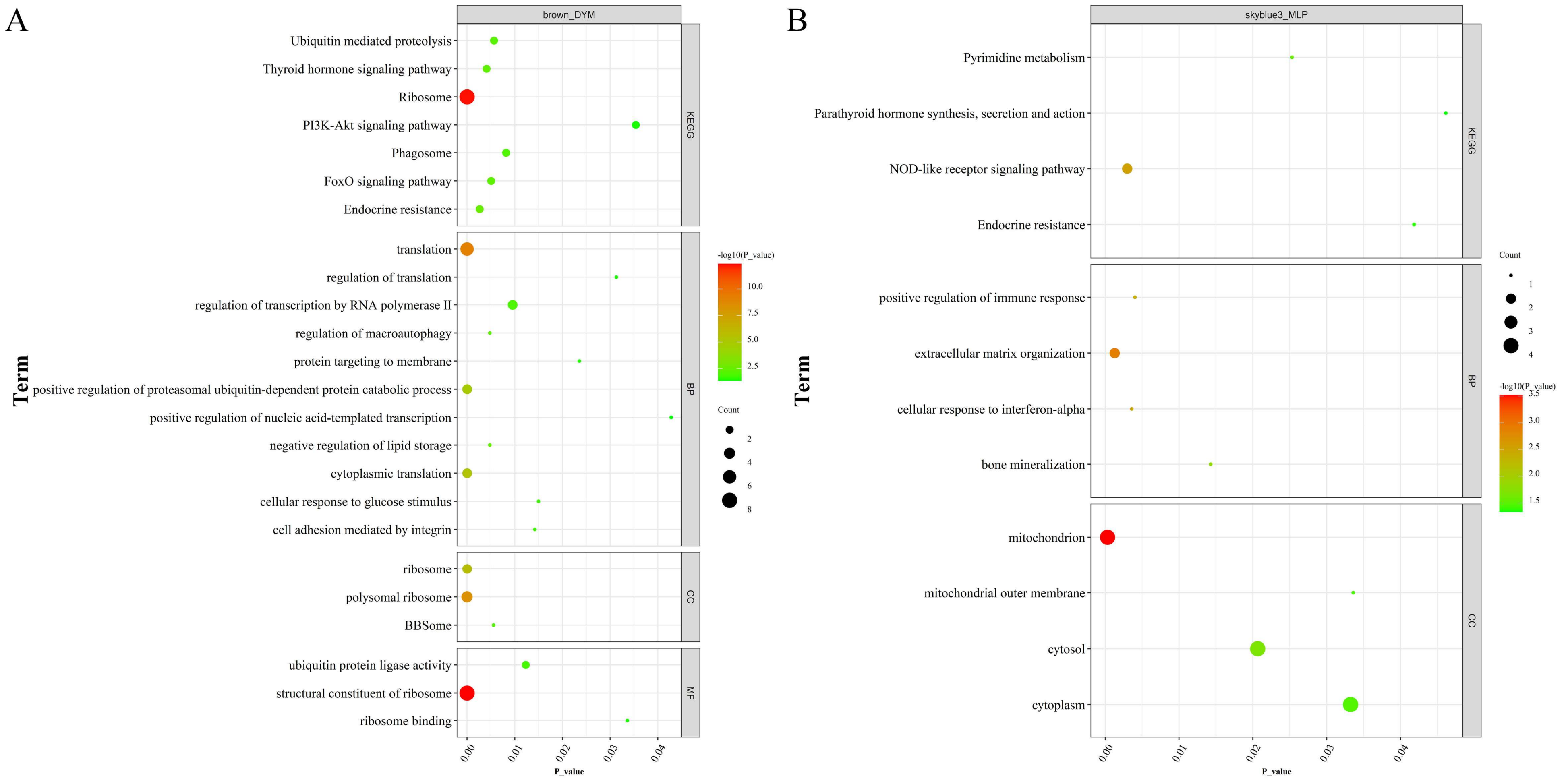
A
brown_DYM
Term
Ubiquitin mediated proteolysis
Thyroid hormone signaling pathway
Ribosome
PI3K-Akt signaling pathway
Phagosome
FoxO signaling pathway
Endocrine resistance
translation
regulation of translation
regulation of transcription by RNA polymerase II
regulation of macroautophagy
protein targeting to membrane
positive regulation of proteasomal ubiquitin-dependent protein catabolic process
positive regulation of nucleic acid-templated transcription
negative regulation of lipid storage
cytoplasmic translation
cellular response to glucose stimulus
cell adhesion mediated by integrin
ribosome
polysomal ribosome
BBSome
ubiquitin protein ligase activity
structural constituent of ribosome
ribosome binding
KEGG
BP
CC
MF
0.00
0.01
0.02
0.03
0.04
P_value
-log10(P_value)
10.0
7.5
5.0
2.5
Count
2
4
6
8
B
skyblue3_MLP
Term
Pyrimidine metabolism
Parathyroid hormone synthesis, secretion and action
NOD-like receptor signaling pathway
Endocrine resistance
positive regulation of immune response
extracellular matrix organization
cellular response to interferon-alpha
bone mineralization
mitochondrion
mitochondrial outer membrane
cytosol
cytoplasm
KEGG
BP
CC
0.00
0.01
0.02
0.03
0.04
P_value
Count
1
2
3
4
-log10(P_value)
3.5
3.0
2.5
2.0
1.5

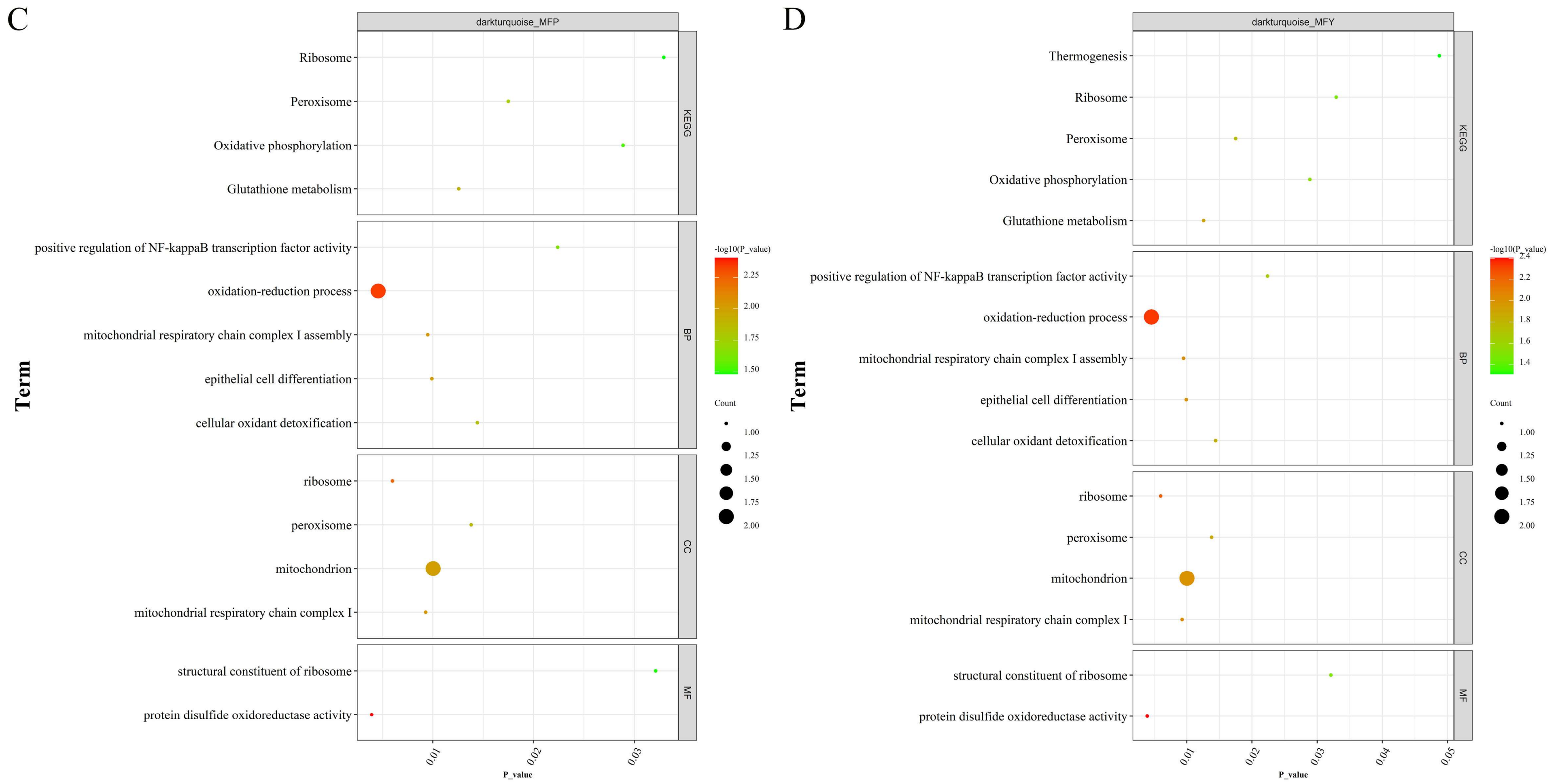
C
darkturquoise_MFP
Ribosome
Peroxisome
Oxidative phosphorylation
Glutathione metabolism
positive regulation of NF-kappaB transcription factor activity
oxidation-reduction process
mitochondrial respiratory chain complex I assembly
epithelial cell differentiation
cellular oxidant detoxification
ribosome
peroxisome
mitochondrion
mitochondrial respiratory chain complex I
structural constituent of ribosome
protein disulfide oxidoreductase activity
KEGG
BP
CC
MF
Term
P_value
0.01
0.02
0.03
-log10(P_value)
2.25
2.00
1.75
1.50
Count
1.00
1.25
1.50
1.75
2.00
D
darkturquoise_MFY
Thermogenesis
Ribosome
Peroxisome
Oxidative phosphorylation
Glutathione metabolism
positive regulation of NF-kappaB transcription factor activity
oxidation-reduction process
mitochondrial respiratory chain complex I assembly
epithelial cell differentiation
cellular oxidant detoxification
ribosome
peroxisome
mitochondrion
mitochondrial respiratory chain complex I
structural constituent of ribosome
protein disulfide oxidoreductase activity
KEGG
BP
CC
MF
Term
P_value
0.01
0.02
0.03
0.04
0.05
-log10(P_value)
2.4
2.2
2.0
1.8
1.6
1.4
Count
1.00
1.25
1.50
1.75
2.00

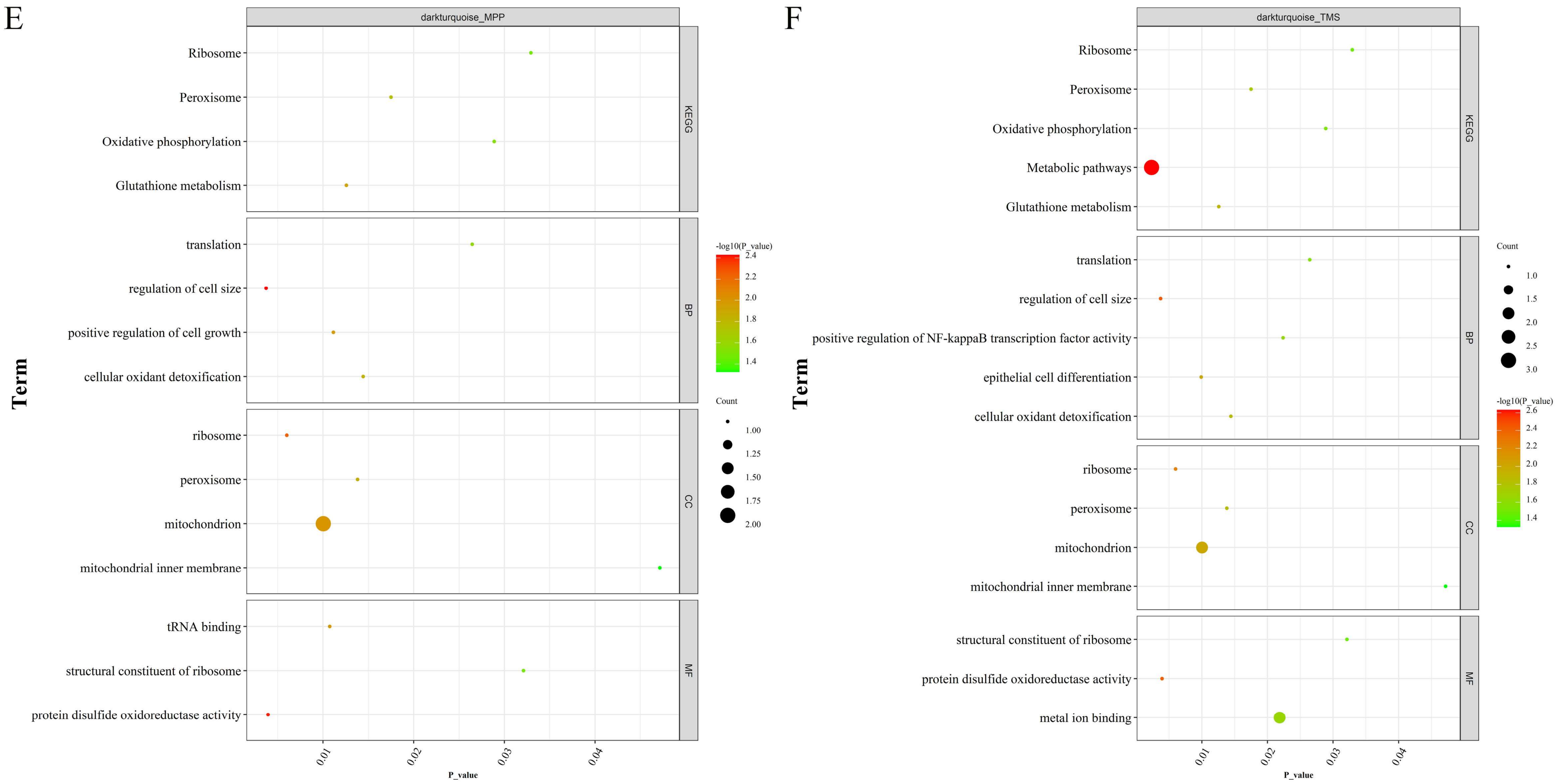

E
darkturquoise_MPP
Ribosome
Peroxisome
Oxidative phosphorylation
Glutathione metabolism
translation
regulation of cell size
positive regulation of cell growth
cellular oxidant detoxification
ribosome
peroxisome
mitochondrion
mitochondrial inner membrane
tRNA binding
structural constituent of ribosome
protein disulfide oxidoreductase activity
KEGG
BP
CC
MF
Term
P_value
0.01
0.02
0.03
0.04
-log10(P_value)
2.4
2.2
2.0
1.8
1.6
1.4
Count
1.00
1.25
1.50
1.75
2.00
F
darkturquoise_TMS
Ribosome
Peroxisome
Oxidative phosphorylation
Metabolic pathways
Glutathione metabolism
translation
regulation of cell size
positive regulation of NF-kappaB transcription factor activity
epithelial cell differentiation
cellular oxidant detoxification
ribosome
peroxisome
mitochondrion
mitochondrial inner membrane
structural constituent of ribosome
protein disulfide oxidoreductase activity
metal ion binding
KEGG
BP
CC
MF
Term
P_value
0.01
0.02
0.03
0.04
Count
1.0
1.5
2.0
2.5
3.0
-log10(P_value)
2.6
2.4
2.2
2.0
1.8
1.6
1.4

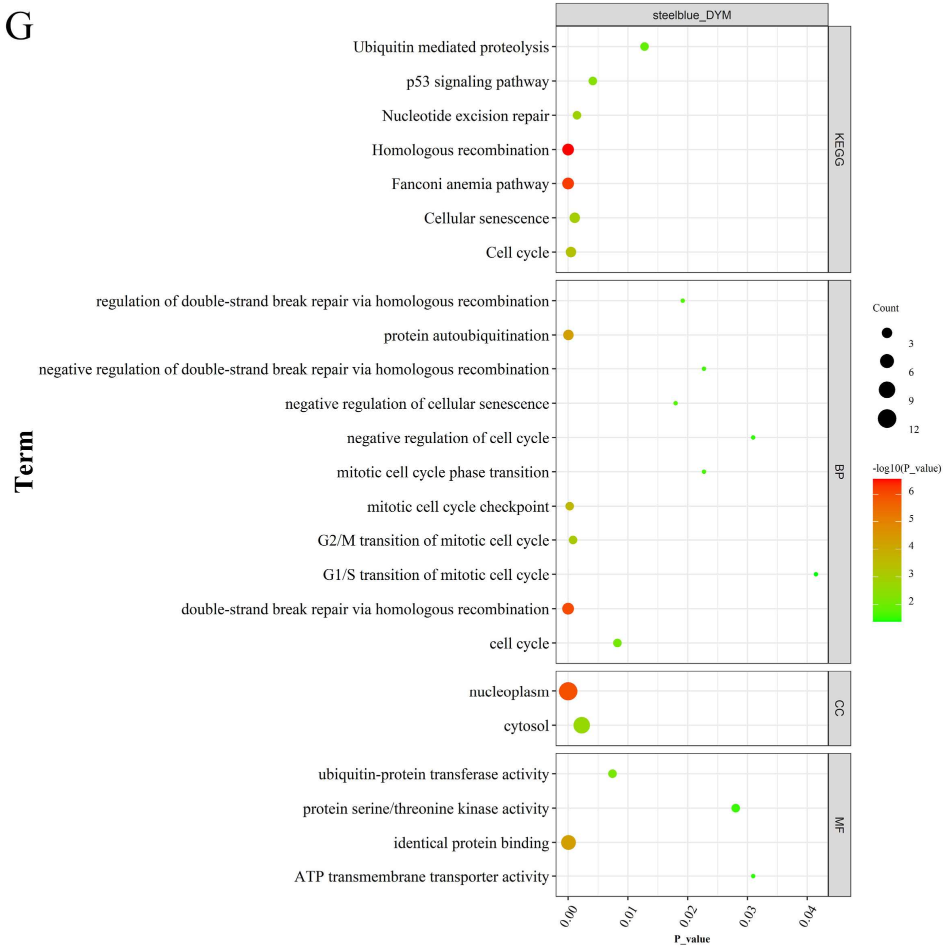

G
steelblue_DYM
Ubiquitin mediated proteolysis
p53 signaling pathway
Nucleotide excision repair
Homologous recombination
Fanconi anemia pathway
Cellular senescence
Cell cycle
regulation of double-strand break repair via homologous recombination
protein autoubiquitination
negative regulation of double-strand break repair via homologous recombination
negative regulation of cellular senescence
negative regulation of cell cycle
mitotic cell cycle phase transition
mitotic cell cycle checkpoint
G2/M transition of mitotic cell cycle
G1/S transition of mitotic cell cycle
double-strand break repair via homologous recombination
cell cycle
nucleoplasm
cytosol
ubiquitin-protein transferase activity
protein serine/threonine kinase activity
identical protein binding
ATP transmembrane transporter activity
KEGG
BP
CC
MF
Term
P_value
Count
-log10(P_value)

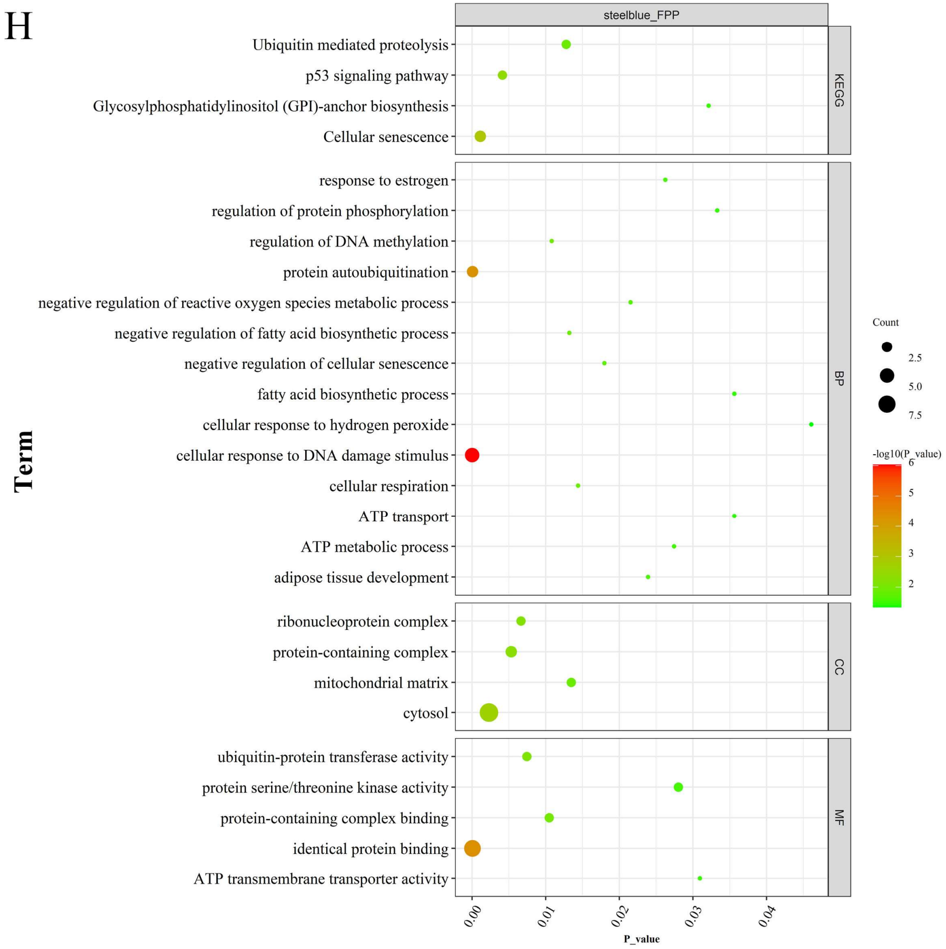

H
steelblue_FPP
Ubiquitin mediated proteolysis
p53 signaling pathway
Glycosylphosphatidylinositol (GPI)-anchor biosynthesis
Cellular senescence
response to estrogen
regulation of protein phosphorylation
regulation of DNA methylation
protein autoubiquitination
negative regulation of reactive oxygen species metabolic process
negative regulation of fatty acid biosynthetic process
negative regulation of cellular senescence
fatty acid biosynthetic process
cellular response to hydrogen peroxide
cellular response to DNA damage stimulus
cellular respiration
ATP transport
ATP metabolic process
adipose tissue development
ribonucleoprotein complex
protein-containing complex
mitochondrial matrix
cytosol
ubiquitin-protein transferase activity
protein serine/threonine kinase activity
protein-containing complex binding
identical protein binding
ATP transmembrane transporter activity
KEGG
BP
CC
MF
Term
P_value
Count
-log10(P_value)

I

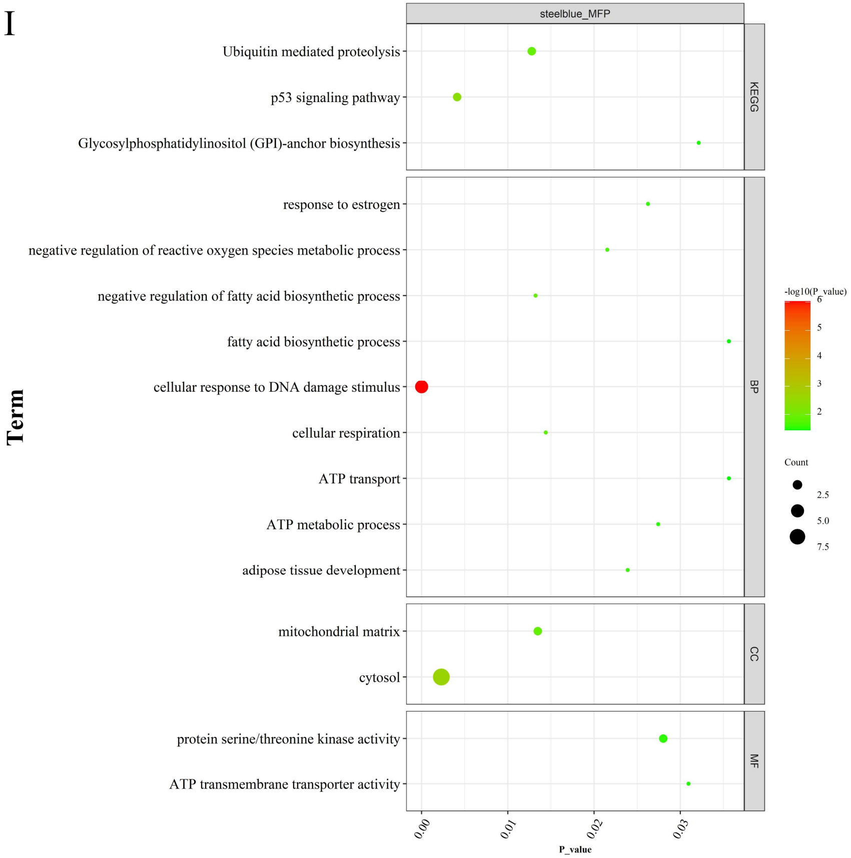

Supplementary Figure S3. Functional enrichment analysis of core genes, the size of the bubble represents the number of enriched genes, the color of the bubble represents the significance, the larger the bubble, the more enriched genes of the pathway/item, the darker the color of the bubble, and the higher the significance. (A) Functional enrichment map of MEbrown core genes on DYM. (B) MLP functional enrichment diagram of MEskyblue3 core gene. (C-F) Functional enrichment diagram of MEdarkturquoise core genes for MFP, MFY, MPP, TMS. (G-I) Functional enrichment diagram of MEsteelblue core genes for DYM, FPP, MFP.

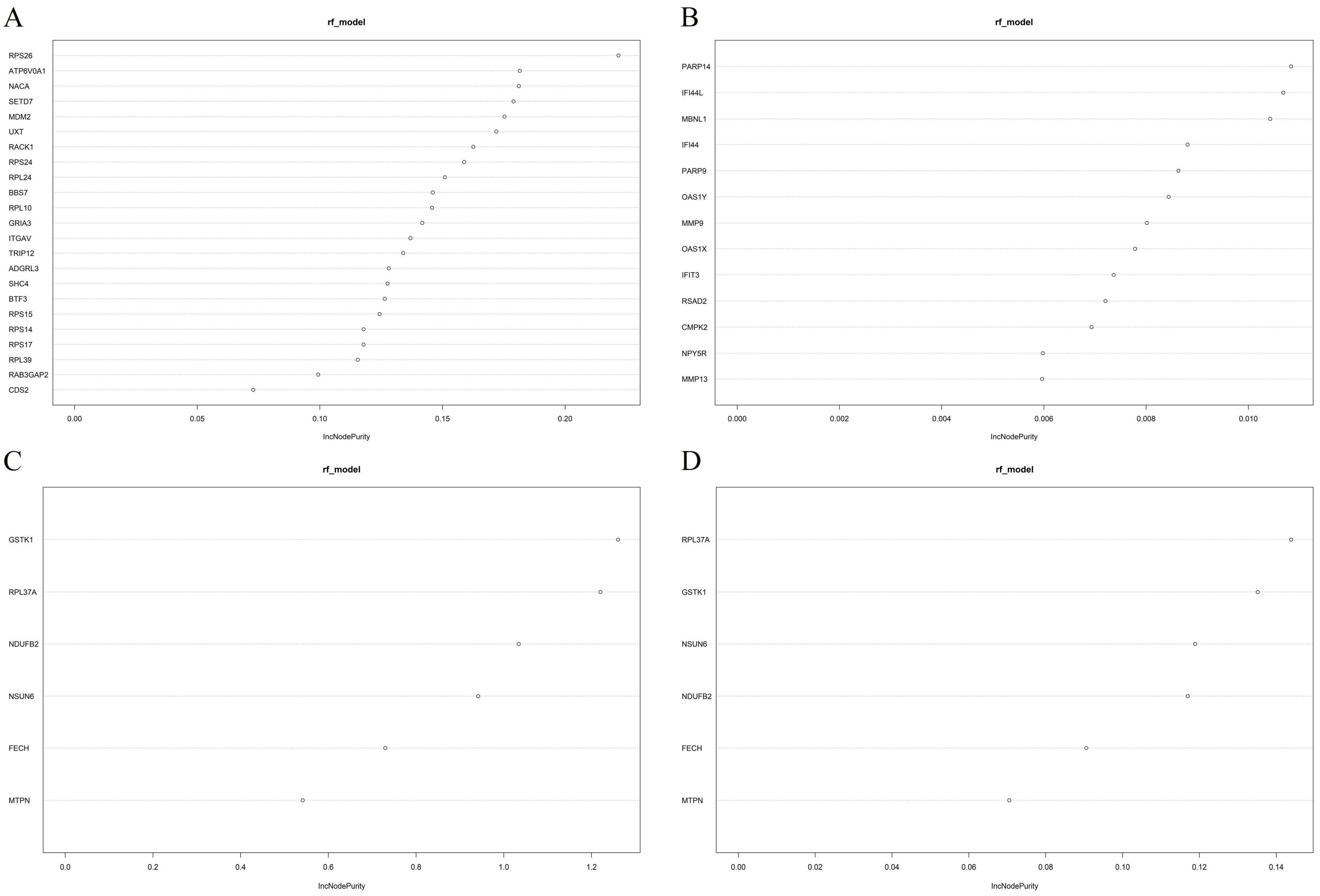

A
rf_model
RPS26
ATP6V0A1
NACA
SETD7
MDM2
UXT
RACK1
RPS24
RPL24
BBS7
RPL10
GRIA3
ITGAV
TRIP12
ADGRL3
SHC4
BTF3
RPS15
RPS14
RPS17
RPL39
RAB3GAP2
CDS2
0.00
0.05
0.10
0.15
0.20
IncNodePurity
B
rf_model
PARP14
IFI44L
MBNL1
IFI44
PARP9
OAS1Y
MMP9
OAS1X
IFIT3
RSAD2
CMPK2
NPY5R
MMP13
0.000
0.002
0.004
0.006
0.008
0.010
IncNodePurity
C
rf_model
GSTK1
RPL37A
NDUFB2
NSUN6
FECH
MTPN
0.0
0.2
0.4
0.6
0.8
1.0
1.2
IncNodePurity
D
rf_model
RPL37A
GSTK1
NSUN6
NDUFB2
FECH
MTPN
0.00
0.02
0.04
0.06
0.08
0.10
0.12
0.14
IncNodePurity

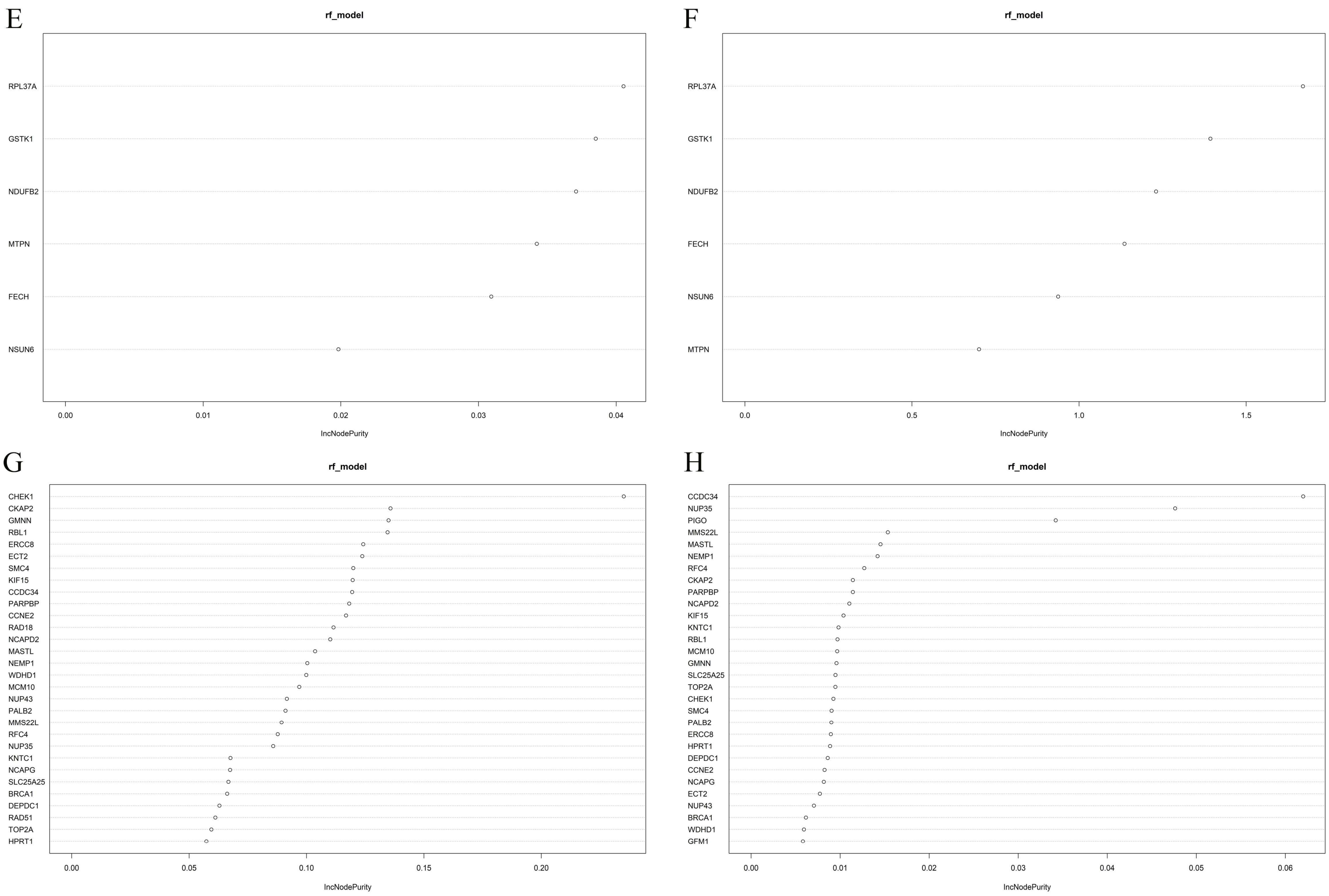
E
rf_model
RPL37A
GSTK1
NDUFB2
MTPN
FECH
NSUN6
0.00
0.01
0.02
0.03
0.04
IncNodePurity
F
rf_model
RPL37A
GSTK1
NDUFB2
FECH
NSUN6
MTPN
0.0
0.5
1.0
1.5
IncNodePurity
G
rf_model
CHEK1
CKAP2
GMNN
RBL1
ERCC8
ECT2
SMC4
KIF15
CCDC34
PARPBP
CCNE2
RAD18
NCAPD2
MASTL
NEMP1
WDHD1
MCM10
NUP43
PALB2
MMS22L
RFC4
NUP35
KNTC1
NCAPG
SLC25A25
BRCA1
DEPDC1
RAD51
TOP2A
HPRT1
0.00
0.05
0.10
0.15
0.20
IncNodePurity
H
rf_model
CCDC34
NUP35
PIGO
MMS22L
MASTL
NEMP1
RFC4
CKAP2
PARPBP
NCAPD2
KIF15
KNTC1
RBL1
MCM10
GMNN
SLC25A25
TOP2A
CHEK1
SMC4
PALB2
ERCC8
HPRT1
DEPDC1
CCNE2
NCAPG
ECT2
NUP43
BRCA1
WDHD1
GFM1
0.00
0.01
0.02
0.03
0.04
0.05
0.06
IncNodePurity

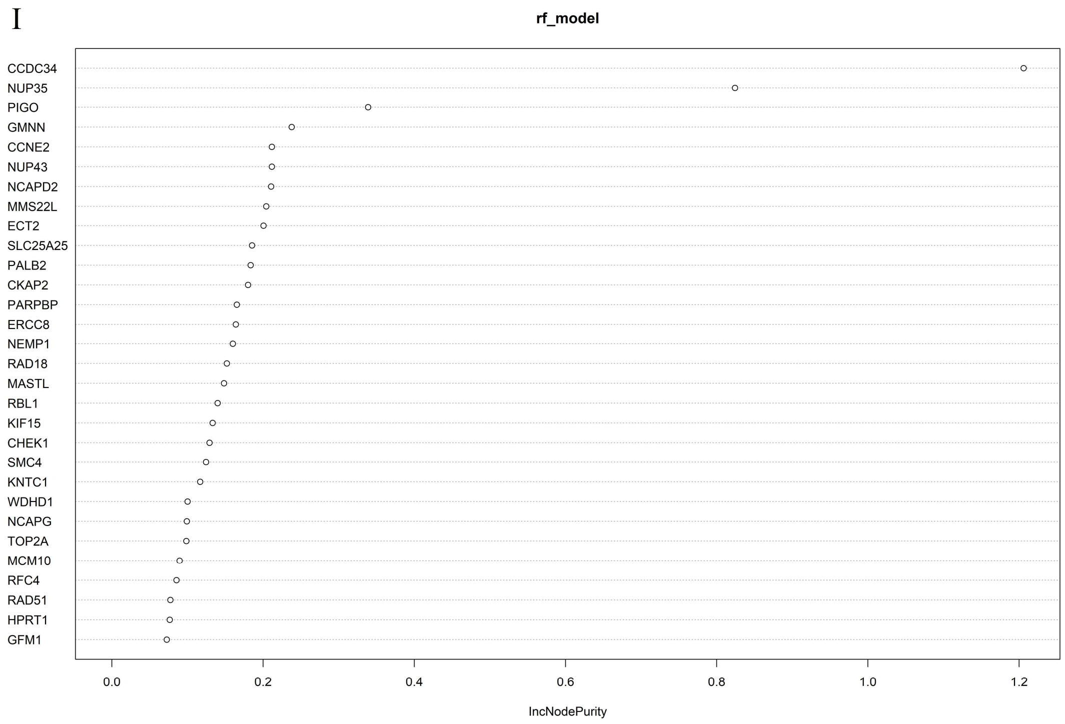

Supplementary Figure S4. Machine-learning score plots for genes in trait-associated modules. (A) Machine-learning scores of genes in the MEbrown module for DYM. (B) Machine-learning scores of genes in the MEskyblue3 module for MLP. (C-F) Machine-learning scores of genes in the MEdarkturquoise module for MFP, MFY, MPP, and TMS, respectively. (G-I) Machine-learning scores of genes in the MEsteelblue module for DYM, FPP, and MFP, respectively.

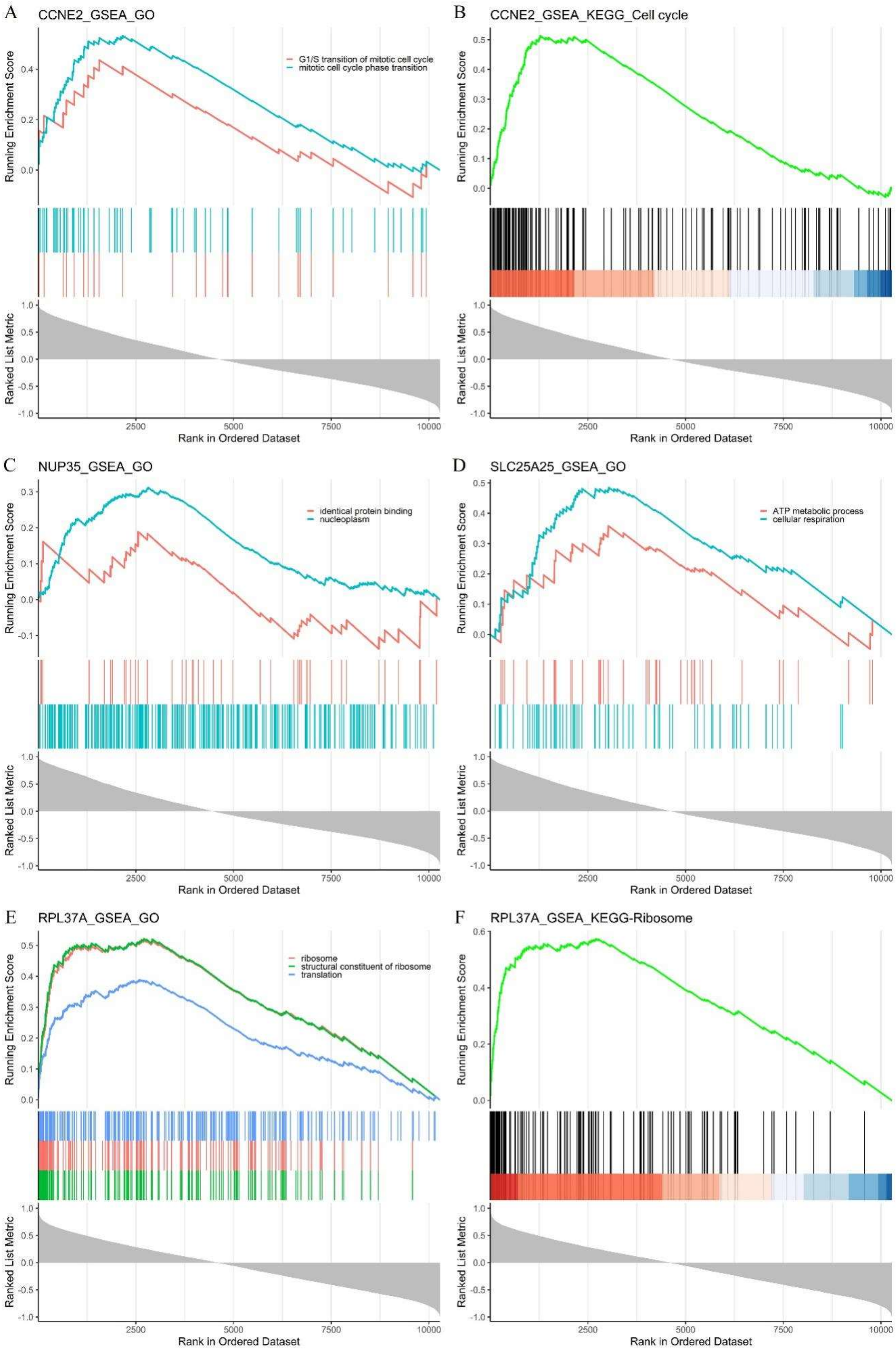

A
CCNE2_GSEA_GO
G1/S transition of mitotic cell cycle
mitotic cell cycle phase transition
B
CCNE2_GSEA_KEGG_Cell cycle
C
NUP35_GSEA_GO
identical protein binding
nucleoplasm
D
SLC25A25_GSEA_GO
ATP metabolic process
cellular respiration
E
RPL37A_GSEA_GO
ribosome
structural constituent of ribosome
translation
F
RPL37A_GSEA_KEGG-Ribosome
Running Enrichment Score
Ranked List Metric
Rank in Ordered Dataset

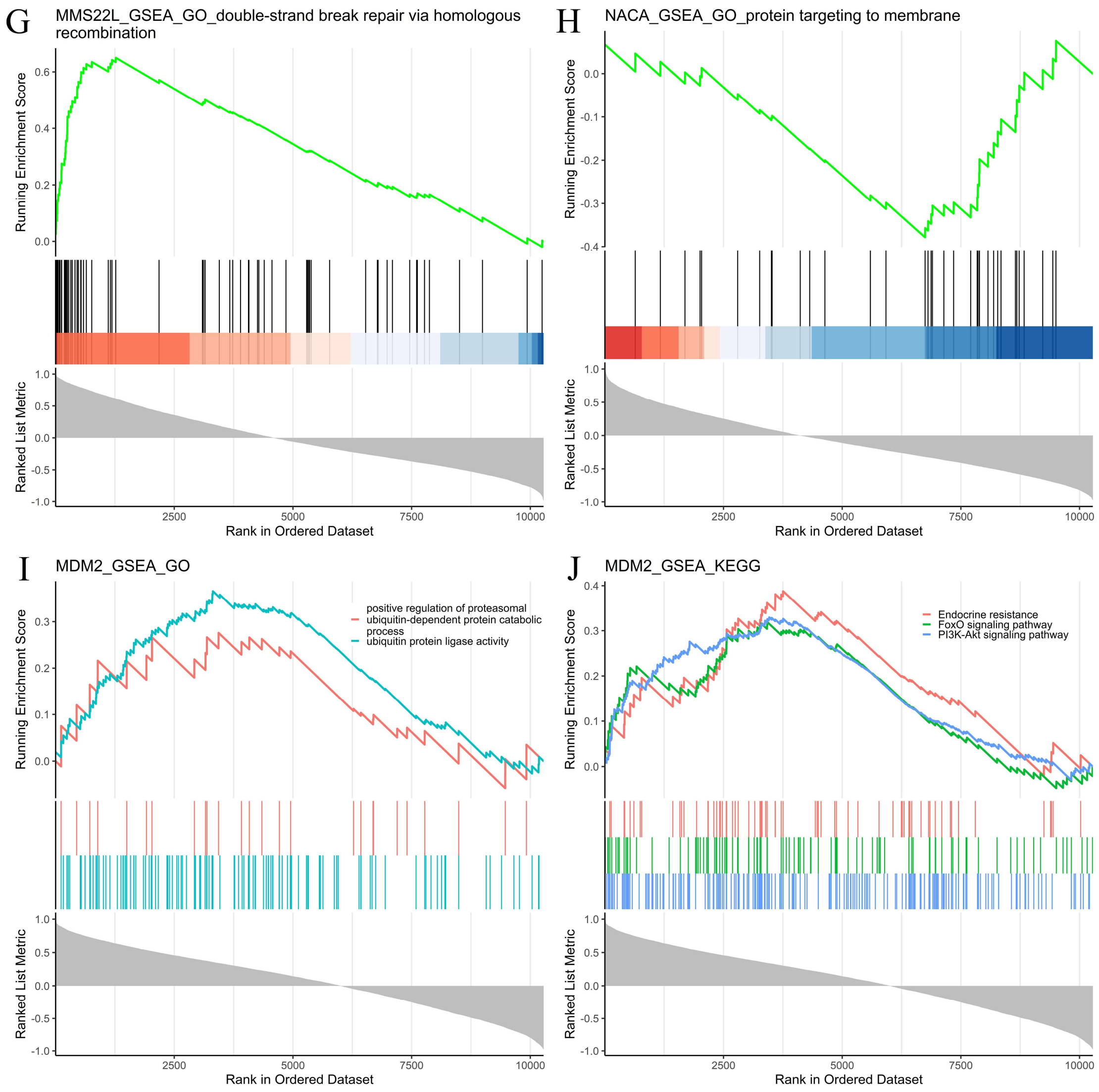

Supplementary Figure S5. Single-gene GSEA plots. The curves show the enrichment of GO terms and KEGG pathways associated with each gene, and the peak indicates the maximum enrichment score. (A,B) GO term and KEGG pathway enrichment plots for *CCNE*2. (C) GO term enrichment plot for *NUP*35. (D) GO term enrichment plot for *SLC25A*25. (E,F) GO term and KEGG pathway enrichment plots for *RPL37A*. (G) GO term enrichment plot for *MMS22L*. (H) GO term enrichment plot for *NACA*. (I,J) GO term and KEGG pathway enrichment plots for *MDM*2.
